# Expanding the phenotypic spectrum of *NAA10*-related neurodevelopmental syndrome and *NAA15*-related neurodevelopmental syndrome

**DOI:** 10.1038/s41431-023-01368-y

**Published:** 2023-05-02

**Authors:** Gholson J. Lyon, Marall Vedaie, Travis Beisheim, Agnes Park, Elaine Marchi, Leah Gottlieb, Tzung-Chien Hsieh, Hannah Klinkhammer, Katherine Sandomirsky, Hanyin Cheng, Lois J. Starr, Isabelle Preddy, Marcellus Tseng, Quan Li, Yu Hu, Kai Wang, Ana Carvalho, Francisco Martinez, Alfonso Caro-Llopis, Maureen Gavin, Karen Amble, Peter Krawitz, Ronen Marmorstein, Ellen Herr-Israel

**Affiliations:** 1 Department of Human Genetics, New York State Institute for Basic Research in Developmental Disabilities, Staten Island, NY USA; 2 George A. Jervis Clinic, New York State Institute for Basic Research in Developmental Disabilities, Staten Island, NY USA; 3 Biology PhD Program, The Graduate Center, The City University of New York, New York, NY USA; 4 Department of Chemistry, University of Pennsylvania, Philadelphia, PA USA; 5 Abramson Family Cancer Research Institute, Perelman School of Medicine, University of Pennsylvania, Philadelphia, PA USA; 6 Institute for Genomic Statistics and Bioinformatics, University Hospital Bonn, Rheinische Friedrich-Wilhelms-Universität Bonn, Bonn, Germany; 7 Institute for Medical Biometry, Informatics and Epidemiology, University Hospital Bonn, Rheinische Friedrich-Wilhelms-Universität Bonn, Bonn, Germany; 8 HematoLogics Inc. Seattle, Seattle, WA USA; 9 Department of Pediatrics, University of Nebraska Medical Center, Omaha, NE USA; 10 Princess Margaret Cancer Centre, University Health Network, University of Toronto, Toronto, ON M5G2C1 Canada; 11 Raymond G. Perelman Center for Cellular and Molecular Therapeutics, Children’s Hospital of Philadelphia, Philadelphia, PA 19104 USA; 12 Department of Medical Genetics, Pediatric Hospital, Coimbra Hospital and University Centre, Coimbra, Portugal; 13 Unidad de Genetica, Hospital Universitario y Politecnico La Fe, 46026 Valencia, Spain; 14 Grupo de Investigacion Traslacional en Genetica, Instituto de Investigacion Sanitaria La Fe, 46026 Valencia, Spain; 15 Department of Biochemistry and Biophysics, Perelman School of Medicine, University of Pennsylvania, Philadelphia, PA USA

**Keywords:** Disease genetics, Medical genetics

## Abstract

Amino-terminal (Nt-) acetylation (NTA) is a common protein modification, affecting 80% of cytosolic proteins in humans. The human essential gene, *NAA10*, encodes for the enzyme NAA10, which is the catalytic subunit in the N-terminal acetyltransferase A (NatA) complex, also including the accessory protein, NAA15. The full spectrum of human genetic variation in this pathway is currently unknown. Here we reveal the genetic landscape of variation in *NAA10* and *NAA15* in humans. Through a genotype-first approach, one clinician interviewed the parents of 56 individuals with *NAA10* variants and 19 individuals with *NAA15* variants, which were added to all known cases (*N* = 106 for *NAA10* and *N* = 66 for *NAA15*). Although there is clinical overlap between the two syndromes, functional assessment demonstrates that the overall level of functioning for the probands with *NAA10* variants is significantly lower than the probands with *NAA15* variants. The phenotypic spectrum includes variable levels of intellectual disability, delayed milestones, autism spectrum disorder, craniofacial dysmorphology, cardiac anomalies, seizures, and visual abnormalities (including cortical visual impairment and microphthalmia). One female with the p.Arg83Cys variant and one female with an *NAA15* frameshift variant both have microphthalmia. The frameshift variants located toward the C-terminal end of *NAA10* have much less impact on overall functioning, whereas the females with the p.Arg83Cys missense in NAA10 have substantial impairment. The overall data are consistent with a phenotypic spectrum for these alleles, involving multiple organ systems, thus revealing the widespread effect of alterations of the NTA pathway in humans.

## Introduction

Targeting 40% of the human proteome, NatA, the major N-terminal acetyltransferase (NAT) complex, acetylates Ser-, Ala-, Gly-, Thr-, Val-, and Cys- N-termini following cleavage of the initiator methionine [1, 2]. The canonical human NatA consists of two main subunits, the catalytic subunit N-α-acetyltransferase 10 (NAA10) (Ard1) and the auxiliary subunit NAA15 (Nat1) and engages with a regulatory subunit, HYPK [3–5]. NAA10-catalyzed N-terminal acetylation has been reported to be essential for development in many species [6–10] and it has been recently shown that mice have a compensating enzyme Naa12, which prevents embryonic lethality in the *Naa10* knockouts [11], but a similar gene has not been found in humans. Furthermore, *NAA10* was also identified in screens for essential genes in human cell lines [12, 13]. Ogden syndrome (OS) was first reported in 2011, and it was given this name due to the location of the first affected family residing in Ogden, Utah, USA [14, 15]. In that first family, with five males dying in early infancy from a range of cardiac and other defects, including lethal cardiac arrhythmias, the pathogenic variant is a single missense change coding for Ser37Pro in the X-linked gene, *NAA10*, which was found in a second independent family in California, USA. The identical variant was recently reported in a third family [16]. There is a *S. cerevisiae* model for the Naa10 Ser37Pro mutant, in which that variant impairs NatA complex formation and leads to a reduction in both NatA catalytic activity and functionality [17, 18]. Furthermore, OS patient-derived cells have impaired in vivo NTA of a few NatA substrates [19].

Since the initial discovery of OS in 2011, multiple groups have reported additional variants either in *NAA10* in both males and females [20–28] or in the heterodimeric protein partner encoded by *NAA15* [21, 29, 30] (and see Supplementary Discussion). Given that NAA10 and NAA15 are part of the NatA complex, it is very likely that there might be shared phenotypes in individuals with pathogenic variants in either of them. As such, the goal of the present study was for one clinician-scientist (G.J.L.) to meet many families with *NAA10* and *NAA15* variants directly and prospectively to uncover patterns that might have been missed by the prior retrospective reviews of medical records and/or summaries provided by the referring clinicians.

## Materials (Subjects) and methods

The methodology is described briefly herein, but further details are in Supplementary Methods. New families were either self-referred or referred by their clinicians. Variants were identified primarily using exome sequencing through clinical diagnostic testing. Molecular effects of the mutations in NAA10 and NAA15 subunits were discussed based upon the human NatA crystal structure (PDB: 6C9M) and the human NatA/HYPK crystal structure (PDB: 6C95), with the heterodimeric NatA crystal structure being used as a representative model for the prepared figures. To validate whether two cohorts share a similar facial phenotype, we conducted a statistical analysis in the clinical face phenotype space of GestaltMatcher [31, 32]. We analyzed 78 NAA10 patients and 33 NAA15 patients, including the patients reported in this work and the previously published patients in the GestaltMatcher Database. The Vineland-3 Comprehensive Interview Form was administered to the parents by one psychologist (E.I.), and provides norm-referenced scores at three levels: subdomains, domains, and the overall Adaptive Behavior Composite (ABC).

## Results

This study involved meeting and assessing 56 individuals/families with *NAA10* variants and 19 individuals/families with *NAA15* variants, which were added to all known cases (*N* = 106 for *NAA10* and *N* = 66 for *NAA15*). All summary data and percentages for phenotype data for these and other published (and some unpublished) *NAA10* and *NAA15* cases was uploaded to the Human Disease Gene website series [33], under the gene names *NAA10* and *NAA15*. The data were downloaded from the website on May 2, 2022; these results are shown in Supplementary Table 1 (an excel file, with tabs for *NAA10* and *NAA15*).

**Fig. 1:**
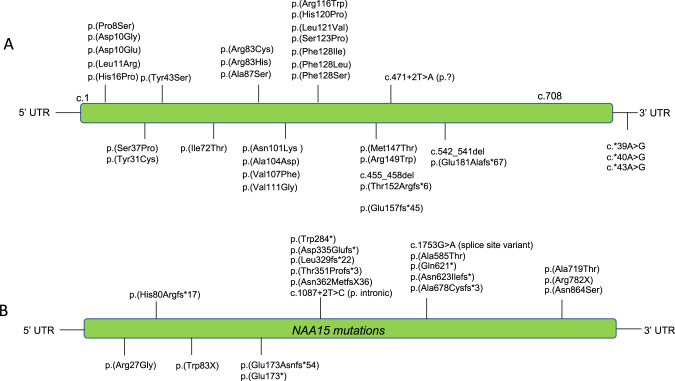
Pathogenic variants in *NAA10* and *NAA15* reported herein. **A** *NAA10* and **B** *NAA15*. UTR untranslated region.

The numbering of individuals and the variants are shown in Fig. 1, Supplementary Table 2 for *NAA10* and in Supplementary Table 3 for *NAA15*. The overwhelming majority of individuals seen were female, where one missense variant (c.247 C > T, p.Arg83Cys) in *NAA10* occurs more frequently and is most recurrent in females, totaling to 25 females seen in this study. However, consistent with this X-linked disease being more severe in males, one male (Individual 9) with the p.Arg83Cys missense in NAA10 died at 11 months of age. Other variants were recurrent, but at a much lower frequency. New information was gathered on every individual reported herein, while a shorter summary was published previously describing some of these individuals with *NAA10* variants (specifically individuals 8, 11, 13–21, 35–38, 41, 47, and 51) [21].

One child (Individual 1) was found to have two de novo variants on the same sequencing reads: [c.22 C > T;30 C > G] p.[Pro8Ser;Asp10Glu]. The c.022 C > T variant affects the first base of exon 2 with possible interference on the splicing of messenger RNA. Clinical studies of the messenger RNA expression in the patient confirmed that mRNA from this allele was not present (Supplementary Fig. 1). The clinical presentation for this child is very similar to many other individuals with other *NAA10* variants, further supporting a haploinsufficiency model in which loss of function of one allele seems to mirror the effects of missense variants in other individuals. Most of the *NAA10* and *NAA15* variants are de novo.

As of April 2022, there are 58 and 64 putative missense or in-frame deletion, substitution or insertion variants in *NAA10* and *NAA15*, respectively, in ClinVar [34], with many of these listed as variants of uncertain significance (Supplementary Table 4). The bioinformatic analyses report consensus classifications for each variant, with an upgrade to pathogenic for several of these.

## Clinical features

Most individuals with variants involving either *NAA10* or *NAA15* have variable degrees of neurodevelopmental disabilities, including impaired motor abilities (HP:0001270), ID (HP:0001249), impaired verbal abilities (HP:0000750) and autism spectrum disorder (ASD) (HP:0000729) (Table 1). These data include previously published cases with variants in *NAA10*, so the overall numbers are greater than just the families that were videoconferenced. Due to having only one X-chromosome, the males with variants in *NAA10* are usually much more severely affected with cardiac issues to the point where some of these individuals died in infancy. However, it is notable that one male, Individual 7 with p.Ile72Thr has no apparent cardiac issues and his level of functioning (as assessed by Vineland-3) was better than some of the females. This is in contrast to a different child (Individual 8) with the exact same variant who experienced a sudden cardiac death around 5 years of age and had prior development of a medulloblastoma [21]. In the cohort, the only other individual developing any type of cancer was Individual 2 with a p.His16Pro missense in NAA10, with leukemia cured by chemotherapy, although a case report of a child with the same exact variant did not report any leukemia or other cancers [20]. As such, this very low level of cancer, with just one case of medulloblastoma and one case of leukemia in this current cohort, could be explained simply by coincidence or other unknown genetic causes not yet identified, but, given the literature regarding the involvement of *NAA10* perhaps in cancer development [35], this likely warrants further investigation.

**Table 1:** Phenotype feature frequency in Individual with *NAA10* variants.

System	Feature	Prevalence (% positive/(negative + unknown)
Neurologic	•Intellectual disability	92/95 (96.8%)
	•Delayed speech	91/95 (95.8%)
	•Motor delay	89/95 (93.7%)
	•Developmental delay	77/95 (81.1%)
	•Hypotonia	57/95 (60.0%)
	•Seizures	34/95 (35.8%)
	•Corpus callosum hypoplasia	27/95 (28.4%)
	•Microcephaly	25/85 (26.3%)
	•Periventricular leukomalacia	17/95 (17.9%)
Psychiatric	•Harming behaviors (self or others)	37/95 (38.9%)
	•Attention deficits	31/85 (32.6%)
	•Eye contact deficits	28/95 (29.5%)
	•Autistic behavior	26/85 (27.4%)
	•Compulsive behavior	16/95 (16.8%)
	•Impulsive behavior	11/95 (11.6%)
Cardiovascular	•Prolonged QT interval	17/95 (17.9%)
	•Atrial septal defects	17/95 (17.9%)
	•Ventricular septal defects	13/95 (13.7%)
	•Hypertrophic cardiomyopathy	9/95 (9.5%)
	•Arrythmias	9/95 (9.5%)
	•Murmurs	8/95 (8.4%)
	•Cardiac arrest	6/95 (6.3%)
Respiratory	•Neonatal respiratory distress	14/95 (14.7%)
	•Pneumonia & other recurrent upper respiratory infections	14/95 (14.7%)
	•Apnea	5/95 (5.3%)
	•Pulmonary hypertension	5/95 (5.3%)
	•Bronchiolitis	4/95 (4.2%)
Gastrointestinal	•Feeding difficulties in infancy	57/95 (60.0%)
	-Gastrostomy tube feeding	12/95 (12.6%)
	-Nasogastric tube feeding	10/95 (10.5%)
	•Feeding difficulties after infancy	40/95 (42.1%)
	•Dysphagia	27/95 (28.4%)
	•Gastroesophageal reflux	21/95 (22.1%)
	•Vomiting	19/95 (20.0%)
	•Bowel incontinence	16/95 (16.8%)
	•Constipation	9/95 (9.5%)
	•Diarrhea	7/95 (7.4%)
Eye abnormalities	•Abnormal eyelashes	42/95 (44.2%)
	•Thick eyebrows	35/95 (36.8%)
	•Abnormal palpebral fissure	22/95 (23.1%)
	•Strabismus	18/95 (18.9%)
	•Hypertelorism	17/95 (17.9%)
Visual abnormalities	•Astigmatism	22/95 (23.1%)
	•Myopia	17/95 (17.9%)
	•Cortical visual impairment	13/95 (13.7%)
Facial features	•Ear abnormalities	50/95 (52.6%)
	-Low-set	32/95 (33.7%)
	-Other outer ear abnormalities	35/95 (36.8%)
	-Middle and internal ear abnormalities	6/95 (6.3%)
	•Nose abnormalities	64/95 (67.4%)
	-Abnormal nasal bridge	39/95 (41.1%)
	-Abnormal nasal tip	48/95 (50.5%)
	-Ala or nares abnormalities	12/95 (12.6%)
	•Mouth abnormalities	69/95 (72.6%)
	-Abnormal philtrum, vermillion	59/95 (62.1%)
	-Abnormal palate	29/95 (30.5%)
	-Teeth and gingival abnormalities	29/95 (30.5%)
	•Chin abnormalities	9/95 (9.5%)
Metabolic & Endocrine	•Sleep disturbance	28/95 (29.5%)
	-Sleep apnea	4/95 (4.2%)
	•Abnormality of temperature regulation	28/95 (29.5%)
	•Slender build	25/95 (26.3%)
	•Hirsutism	14/95 (14.7%)
	•Precocious puberty	6/95 (6.3%)

**Fig. 2:**
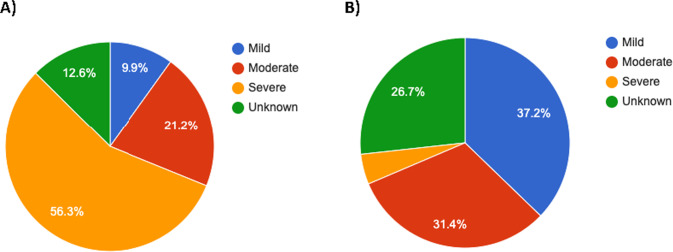
Severity of Intellectual Disability. **A** Individuals with *NAA10* variants (*n* = 95); **B** Individuals with *NAA15* variants (*n* = 57).

Various levels of ID are reported in almost all study subjects with available data, including mild, moderate or severe ID, and learning difficulties with or without behavioral issues. The ID is usually much more severe for NAA10 than *NAA15* cases (Fig. 2), which is once again consistent with X-linked inheritance for *NAA10*, along with the fact that NAA10 is the acetyltransferase enzyme, whereas NAA15 is the dimeric binding partner that helps to localize the NatA complex to the ribosome. Overall, individuals with *NAA10* variants more often have much more impaired motor function, including fine motor difficulties, abnormality of movement, motor delay, and hypotonia, in comparison to individuals with *NAA15* variants.

The two male individuals with frameshift variants in *NAA10* (Individuals 55 and 56) are generally much higher functioning than the other individuals with missense variants (see Supplementary Information). These frameshift variants occur toward the C-terminus of the protein, with Individual 56 being the most C-terminal with Glu181Alafs*67 in *NAA10*. Therefore, it is likely that some functional N-terminal acetyltransferase enzymatic activity remains.

Most individuals with *NAA10* variants have verbal issues including complete absence of speech, delayed language development, required use of sign language, or other speech difficulties related to articulation, which some parents report as possibly being connected to low muscle tone in the pharyngeal muscles. Some research participants also present with either ASD and/or other behavioral challenges. The birth weight was low (≤5th percentile) in a few individuals and it was noted that some individuals failed to grow, causing their weights to fall below the 5th percentile over time. The percentages for some clinical features are listed in Table 1.

The most notable new information relates primarily to the development and maturation of these individuals, as it has become apparent that there are several hurdles to overcome during development, and that failure to overcome each hurdle significantly impacts the trajectory of development. First, these individuals must overcome the neonatal lethality of possible cardiac arrythmias and cardiovascular or respiratory complications. This seems to be more common in males, but one female (Individual 3) with a p.Tyr31Cys missense in NAA10 died at 6.5 months with such issues after spending some time in the neonatal intensive care unit (NICU), which also occurred with male infants in the past, including the male Individual 9 reported herein. The second hurdle involves feeding difficulties in infancy including swallowing food and liquids, as some individuals have severe hypotonia, and ultimately require feeding tubes [36]. The third hurdle involves remaining seizure-free, as it seems that the cognitive development of the individuals is markedly delayed if seizures begin and are not well-controlled. Lastly, the fourth hurdle involves learning to walk, as some of the individuals remain wheelchair-bound, due to a lack of coordination and/or an inability to stay balanced when walking, which seems to also be related to low muscle tone. As such, the clinical presentation can be quite variable, as someone who requires tube-feeding, is wheelchair bound, and is suffering from seizures will likely have a far different cognitive outcome than someone who does not experience these setbacks.

## Facial features

There are commonly seen and sometimes recognizable traits in individuals with *NAA10* variants, including thicker eyebrows, long eyelashes, upturned nose, and broad nasal bridge (Table 1, Supplementary Figs. 2–4). While the facial features in the males and females can also be quite variable, some features, including an upturned nose, along with bushy eyebrows and long eyelashes, are quite common. Other possible features include delayed closing of anterior and posterior fontanels, presence of a broad and/or prominent forehead, and sparse anterior scalp hair. Facial feature abnormalities also vary in many individuals with *NAA15* variants (Supplementary Fig. 5). The most common are abnormalities of the eyebrows (horizontal, thick, or long), long eyelashes, hypertelorism, prominent epicanthal folds, abnormalities of the palpebral fissure, amblyopia, astigmatism, and/or strabismus.

To investigate further the similarity between the two cohorts based on facial gestalt, we conducted a facial analysis using the GestaltMatcher approach [31, 32], using all available facial photographs for *NAA10* and *NAA15* patients, including previously published cases (Supplementary Table 5). Supplementary Fig. 7 shows that *NAA10* and *NAA15* patients do not separate clearly. Moreover, comparing the cohorts *NAA10* and NAA15 in the clinical face phenotype space resulted in a mean pairwise cosine distance of *d* (*NAA*10, *NAA*15) = 0.9146 between two patients. The mean pairwise distance falls below the threshold *c* = 0.9176 defined to decide whether two cohorts stem from the same syndrome (mean pairwise cosine distance smaller than *c*) or from two different syndromes (mean pairwise cosine distance greater than *c*). A subsampling approach yielded that 67% of sampled subcohorts showed similar phenotypes. Overall, our analysis indicates an overlap in the facial gestalt of NAA10 patients and NAA15 patients (Supplementary Fig. 8).

## Cardiovascular features

The majority of females and even some males have no obvious cardiac issues. However, several of the males had structural anomalies of their hearts, including ventricular septal defect, atrial septal defect, and pulmonary artery stenosis. For those who died, arrhythmias at the time of death included torsade de pointes, premature ventricular contraction (PVC), premature atrial contraction (PAC), supraventricular tachycardia (SVtach), and ventricular tachycardia (Vtach). EKG analysis of OS patients has demonstrated a large proportion of patients demonstrating an elongated QT interval congruent with previously published literature [37]. Our data shows an equal proportion of patients with elongated QT intervals as patients with atrial septal defects in patients with variants in *NAA10* (Table 1).

## Respiratory features

The vast majority of probands do not have any major respiratory issues, but there are a few isolated cases of interstitial lung disease and/or respiratory distress (Table 1). For example, Individual 3 with p.Tyr31Cys missense in NAA10 died in the first year of life with local interstitial lung disease, neonatal respiratory distress, respiratory failure requiring assisted ventilation, lymphangiectasia, and pulmonary fibrosis. A lung biopsy reported mild thickening of the interstitium and focal interstitial glycogenosis confirmed with PAS staining. The Individual 9, a male with p.Arg83Cys missense in NAA10, died around 1 year of age, also with respiratory arrest, neonatal respiratory distress, respiratory failure requiring assisted ventilation, tachypnea, and interstitial lung disease. The original OS males also had respiratory distress and frequent pneumonias [14], seen also in a third family [16], but these other individuals without primary cardiac arrhythmias seem to demonstrate that this respiratory distress can be primarily caused by interstitial lung disease, rather than being entirely secondary to cardiovascular complications.

## Gastrointestinal features

The gastrointestinal pathology associated with OS, in order from most to least prevalent, includes feeding difficulties in infancy, dysphagia, GERD/silent reflux, vomiting, constipation, diarrhea, bowel incontinence, and presence of eosinophils on esophageal endoscopy. The percentages for some of these are shown in Table 1 and/or are available on the Human Disease Gene website series. In addition, the gastrointestinal symptom profile for individuals with this syndrome has been expanded to include eosinophilic esophagitis, cyclic vomiting syndrome, Mallory Weiss tears, abdominal migraine, esophageal dilation, and subglottic stenosis [36]. A recent analysis including nine G-tube or GJ-tube fed probands demonstrated that G/GJ-tubes are overall efficacious with respect to improvements in weight gain and caregiving [36].

## Growth, including height, weight and head circumference

Many of the OS individuals and a few of the individuals with *NAA15* variants have low height and weight, as detailed in a recent study [36]. Poor growth cannot be entirely explained by inadequate caloric intake, inability to properly chew or swallow, growth hormone deficiency, or low appetite. Past the age of 6-12 months, calorie tracking, caloric supplementation, and using feeding therapy to successfully teach individuals how to chew, swallow, and no longer choke on food did not induce adequate weight gain. Low appetite was also not a consistent cause of poor growth, as multiple proband parents claimed that their individuals had great appetites. Growth hormone deficiency was treated with growth hormone (GH) administration in two probands but these efforts kept these probands in the failure to thrive (FTT) range, despite some slight weight improvements.

## Endocrine and metabolic features

Some of these features are shown in Table 1. Extensive laboratory panels assessing digestive function, screening for metabolic decompensation, and hormonal dysregulation have not been significantly useful in improving the care or outcome for these individuals. Although some parents report that their children are quite lean with thermoregulation abnormalities, there is no metabolic or quantitative data yet to prove this claim. Although evidence from mouse models [38] indicates dysregulation of glucose and insulin levels, the one human proband (Individual 46) tested thus far with extensive glucose monitoring over 14 days did not show any major abnormalities. Mouse models also show decreased brown adipose tissue, which might offer an explanation for the thermoregulation issues [38], but brown adipose tissue amounts have not yet been assessed in affected individuals. There are occasional findings in particular individuals that may or may not be related to the variants in *NAA10* or *NAA15*. For example, Individual 8 with an *NAA15* variant has been found to have corneal calcifications and persistent serum hypercalcemia, although there is no other laboratory-based evidence for hyperparathyroidism.

## Ophthalmologic and visual features

The majority of *NAA10* individuals present with visual abnormalities, which often include astigmatism, myopia, strabismus, and/or amblyopia (also see Table 1). Further, there are a subset of individuals who have exotropia/esotropia, microphthalmia, are blind, and/or have abnormalities to the optic disc or nerve. In *NAA15* individuals, visual abnormalities were present in a small subset as strabismus, amblyopia, astigmatism, and other visual impairment. Many individuals with this syndrome wear eyeglasses and due to this high prevalence of visual impairment, eye examination by ophthalmology is warranted. Further, thirteen individuals with *NAA10* variants and one male with p.Asn864Ser in NAA15 presented with cortical or cerebral visual impairment (CVI). CVI can be described as a “verifiable visual dysfunction which cannot be attributed to disorders of the anterior visual pathways or any potentially co-occurring ocular impairment” [39].

Individual 23 is a female with the common p.Arg83Cys missense in *NAA10*, yet she is the only female published to date who was born with microphthalmia, with a very small right eyeball along with a smaller, misshapen pupil, leading to the need for a prosthetic eyeball (see Supplementary Fig. 2). She also has stigmatism in her left eye, along with myopia and possible cortical visual impairment. Her exome sequencing did not reveal any other possible cause for the microphthalmia. Individual 54 (a male) with p.Thr152Argfs*6 in *NAA10* also has microphthalmia (see Supplementary Fig. 3), similarly to a previously published case [21], whereas the male in Japan did not have microphthalmia [40].

After the posting of an earlier version of this manuscript as a preprint [41], one clinician (A.C.) reported to us the discovery of one female (19 years old) with unilateral microphthalmia with a *NAA15* frameshift variant. This is further proof that the expression of many phenotypes is likely titrated by the overall amounts of monomeric NAA10 and/or NatA activity, whereby microphthalmia occurs in both syndromes when some threshold is crossed during development in which likely some substrate is not acetylated enough.

## Neurologic features and recommendations

The prototypical subject displays intellectual disability, global developmental delays, gross and/or fine motor delay, hypotonia, and speech delay. In many cases speech delay was severe enough for speech to be considered absent. In addition, the presence of tonic-clonic seizures was not uncommon. Virtually all individuals have neonatal hypotonia, and several had neurogenic scoliosis. For Individual 45, intervention for neurogenic scoliosis was attempted with placement of rods. The percentages for some of the neurologic features are shown in Table 1.

Neuroimaging has been obtained in a number of these individuals and there is a paper in preparation regarding detailed analysis of brain MRI findings. The majority possessed at least one brain abnormality with corpus callosum hypoplasia and microcephaly being by far the most common findings followed by periventricular leukomalacia (see Supplementary Table 1 for exact figures). In some cases, there was a finding of cerebral atrophy. However, there was no radiographic evidence to explain their hypotonia or growth failure.

## Cognitive and psychiatric features

**Fig. 3:**
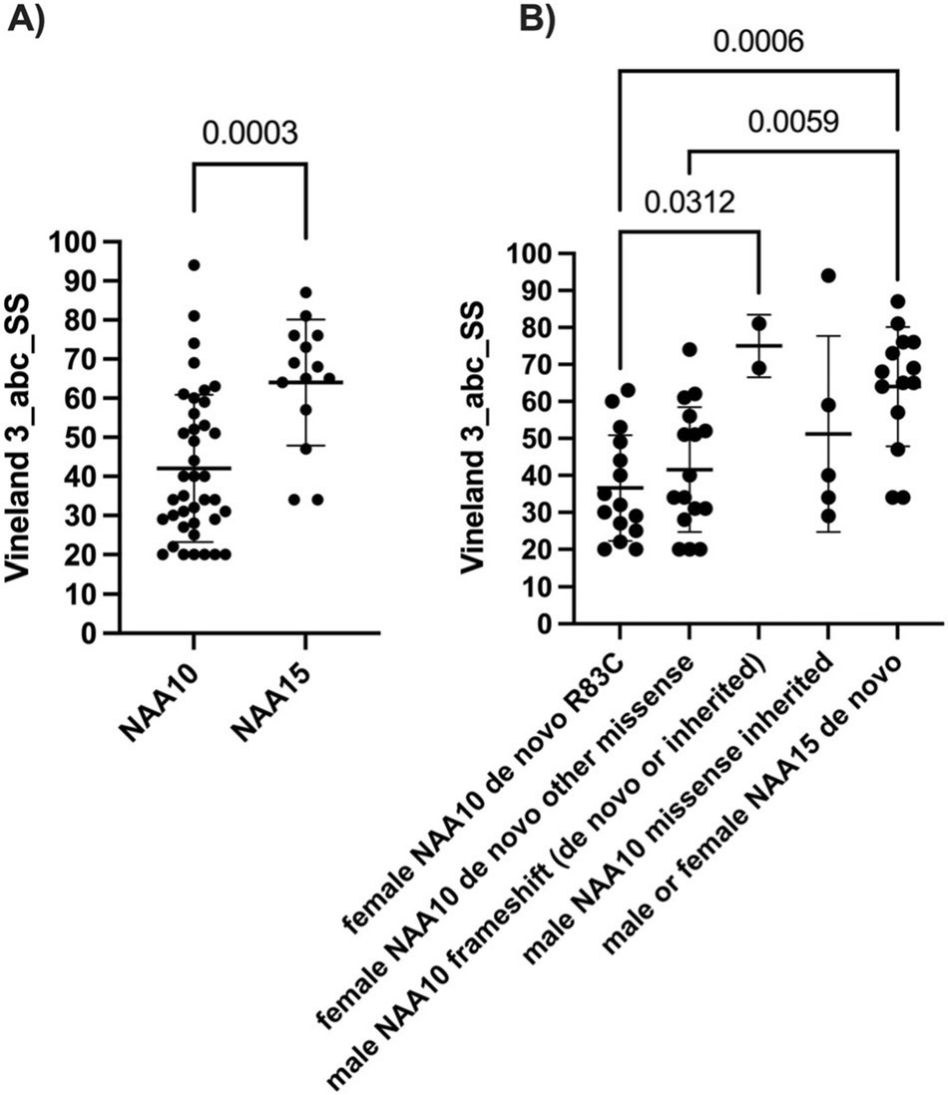
Vineland results. Adaptive Behavior Composite (ABC) Standard scores have a mean of 100 in the “normal” population and SD of 15. **A** All scores are plotted for *NAA10* and *NAA15*, without regard for sex, inheritance pattern, or type of variant. **B** These same scores are now separated out by sex, inheritance pattern and type of variant. In the case of *NAA15*, which is an autosomal gene, the sexes are still grouped together.

The psychiatric profile of subjects with OS typically includes harmful behavior which is either outwardly directed (agitation or violent behavior toward others) or inwardly directed/self-injurious (hair pulling, self-biting, head-banging, etc) in addition to impulsive or compulsive behavior. Short attention span, poor eye contact, and autistic, stereotyped or repetitive behaviors were common (Table 1). Vineland-3 assessment revealed substantial variability, although the overall level of functioning for the *NAA15* probands was significantly higher than the *NAA10* probands (Fig. 3A, Supplementary Table 6), consistent with more *NAA10* probands having a “severe” intellectual disability diagnosis (Fig. 2). When the results for NAA10 were separated by sex, inheritance pattern, and variant type, it became clear that the frameshift variants located toward the C-terminal end of NAA10 have much less impact on overall functioning. For the females with the p.Arg83Cys missense in NAA10, there is also substantial phenotypic variability with functioning as well, which appears to be equivalent in scope to the many other variants in females (Fig. 3B). As such, there are clearly other factors affecting the overall trajectory of these individuals.

## Human phenotype ontology-based clustering analyses

Hierarchical clustering analyses were performed using the HPO terms downloaded from the Human Disease Gene website series in October 2022 (see Supplementary Table 1). The two syndromes for *NAA10* and *NAA15* do cluster separately, albeit with some overlap (see Supplementary Fig. 6). Most importantly, and consistent with the clinical impression of these two syndromes, there was no obvious sub-clustering among the different variants within each syndrome, providing quantitative support for a phenotypic spectrum within each syndrome, with overlap between them.

## Molecular analysis of the variants

Most *NAA10* variants identified to date in *NAA10*-related neurodevelopmental diseases reduce NAA10- and NatA-type activity [21]. Although novel variants clinically described here have not been characterized biochemically, it is possible to anticipate biochemical implications resulting from variants in the conserved region of NAA10 (residues 1-160) based on previous studies[14, 17, 19–22, 24, 42, 43]. However, variants and truncations occurring in the C-terminus of NAA10 require additional studies to understand the role of the NAA10 C-terminal domain.

**Fig. 4:**
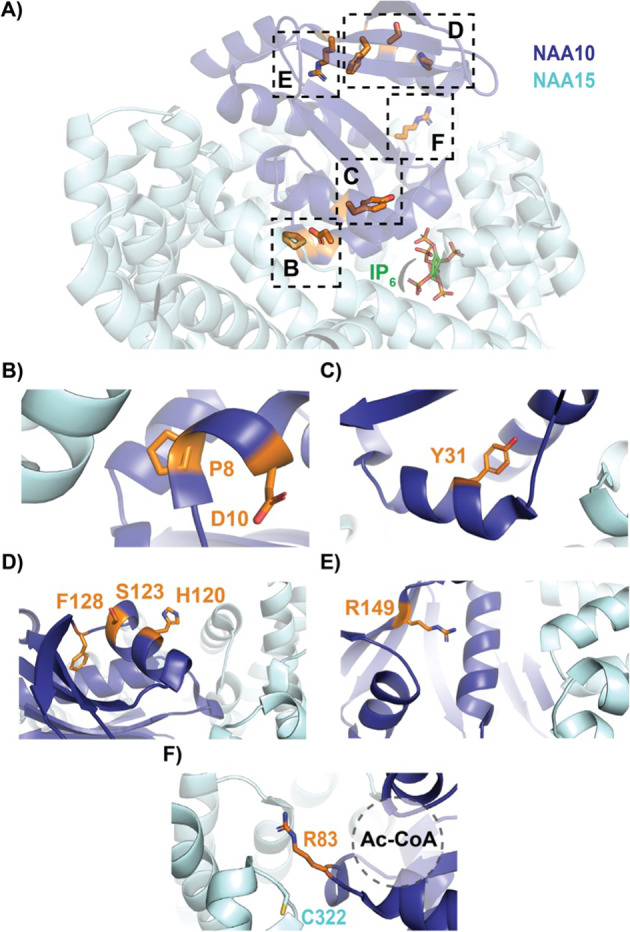
Crystal structure of human NaA complex labeled with relevant NAA10 residues. NAA10 (dark blue) and NAA15 (light cyan) are shown in cartoon with wild-type residues in stick (orange). **A** Top view of complex annotated with relevant NAA10 residues outlined by dashed boxes: (**B**) P8 and D10; **C** Y31; **D** H120, S123, and F128; **E** R149; and **F** R83 and NAA15 C322 (light cyan, stick), where the acetyl CoA binding pocket is indicated by a dash-outlined circle.

To contextualize the phenotypes associated with the NAA10 and NAA15 mutant proteins, we inspected two published human NatA crystal structures: the heterodimeric complex composed of the NAA10 and NAA15 subunits (PDB: 6C9M) and the heterotrimeric complex composed of NAA10, NAA15, and the regulatory subunit, HYPK (PDB: 6C95). Based on these structures, the *NAA10* variants p.Pro8Ser/Asp10Glu, p.Tyr31Cys, p.His120Pro, p.Ser123Pro, p.Phe128Ser, and p.Arg149Trp would likely compromise the folding and/or thermal stability of NAA10 (Fig. 4) [5]. Specifically, the mutations to proline (p.His120Pro, p.Ser123Pro) occur in helices, likely destabilizing the helical structure, and the p.Tyr31Cys and p.Phe128Ser mutations likely disrupt hydrophobic core interactions that are mediated by Tyr31 and Phe128.

The biochemistry of the p.Arg83Cys variant is unique amongst the NAA10-type variants. The monomeric NAA10 p.Arg83Cys protein is less active than wild type NAA10 [43] and the heterodimeric p.Arg83Cys NatA complex was found have enhanced activity, while HYPK binding returns p.Arg83Cys NatA activity back to wild type levels [21]. In the context of the heterodimeric NatA complex, the binding of acetyl CoA to p.Arg83Cys NAA10 is compromised, as indicated by the loss of acetyl CoA-mediated stabilization [21]. For the wild type NAA10, the positively charged p.Arg83Cys side chain interacts with the negatively charged acetyl CoA pantothenic arm, which would be absent with the Cys variant. In addition, it is possible that this complex NatA phenotype arises from the formation of a disulfide bond between NAA10 p.Arg83Cys with the nearby NAA15 p.Cys322 residue. This disulfide bond has been proposed to anchor the complex so that the complex is better positioned to catalyze the N-terminal acetylation reaction. This disulfide bond would not form in the monomeric NAA10 state, rendering mutant NAA10 p.Arg83Cys less active than the wild type monomeric NAA10.

In contrast to the reported *NAA10* variants, *NAA15* variants tend to confer a range of biochemical and biophysical effects on NatA function [21]. Similar to *NAA10* variants, several novel variants have been included that have not yet been biochemically characterized (p.Arg27Gly, p.Ala585Thr, p.Ala719Thr, and p.Asn864Ser). Variants occurring in NAA15 could affect NAA15 folding and/or thermostability, as well as intermolecular interactions with NAA10 (directly or through a bridging inositol hexaphosphate, IP_6_, molecule) or HYPK—and, thereby, NatA catalytic activity, or NatA localization to the ribosome.

**Fig. 5:**
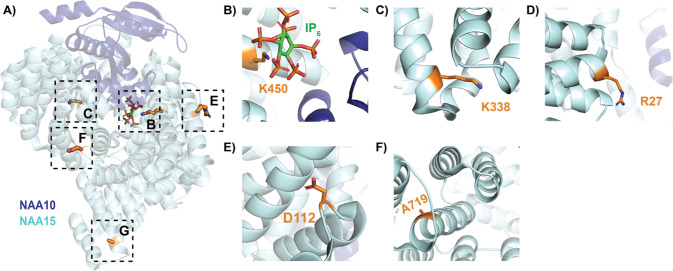
Crystal structure of human NatA complex labeled with relevant NAA15 residues. NAA10 (dark blue) and NAA15 (light cyan) are shown in cartoon with wild-type residues in stick (orange). **A** Side view of complex annotated with relevant NAA15 residues outlined by dashed boxes: (**B**) K450 with bound inositol hexaphosphate (IP_6_, stick); **C** K338N; **D** R27; **E** D112; and **F** A719.

Based on structural considerations of the human NatA and NatA/HYPK crystal structures (Fig. 5) and previous in vitro studies, [21] we anticipate that the NAA15 p.Lys450Glu mutant would disrupt the NAA10-IP_6_ interaction and, thus, destabilize the NAA10/NAA15 complex, while the p.Lys338Asn mutant could disrupt NAA15 thermostability by disrupting a hydrogen bond between two adjacent helices in NAA15. p.Ala585Thr is disordered in both crystal structures and mutants p.Arg27Gly and p.Asp112Asn are solvent-exposed on NAA15, so it is unclear how these mutations impact NatA function. However, it is possible that they disrupt the NatA-ribosome interaction. In particular, p.Arg27 and p.Ala585 are both located in regions previously implicated in ribosome-binding, thereby altering NatA co-translational activity [44]. P.Ala719Thr is buried within a bundle of helices in the metazoan-conserved NAA15 C-terminus, which is critical for NatA heterodimer stabilization and HYPK regulatory subunit binding [5]. Finally, the molecular basis for the p.Asn864Ser mutant is unclear because published NatA structures are not resolved past residue 841.

## Discussion

We document an extensive phenotypic spectrum caused by likely decreased expression and/or function of NAA10 and/or the NatA complex, with the cases involving *NAA10* variants usually presenting as more severe than cases involving *NAA15*. Mendelian diseases are anything but simple, as the presentations can be quite complex and variable due to modification by genetic background and environmental influences [45]. The different trajectories are impacted by whether the child overcomes certain hurdles, such as learning to feed themselves and to walk.

One limitation of our study is that only the first nine research participants were interviewed in-person and physically examined by medical doctors, with all interviews converted to videoconferencing starting in March 2020, with the onset of the COVID19 pandemic. However, we did not observe any major differences in the information obtained from the in-person visits as compared to the videoconference visits, and all visits included review of available medical records. Please see the Supplementary Discussion for additional details.

To date, most NAA10 and NAA15 variants have been studied in vitro using either immunopurified overexpressed full-length NAA10 (235 residues) protein from yeast [17] or mammalian culture [20, 22, 24], *S. frugiperda* (*Sf*9)-expressed recombinant NatA complex containing a C-terminally truncated NAA10 (1-160) [21], or an affinity-tagged full-length NAA10 expressed in *E. coli* [14, 19, 43]. Each of these methodologies have their limitations (see Supplementary Discussion), and these in vitro assays are also limited in scope as there could very well be cellular and organismal phenotypes that result from decreased (or otherwise altered) amino-terminal acetylation of a wide range of substrates.

Although some papers have also suggested that there might be different allelic presentations or mechanisms of action for different variants involving *NAA10*, such as with microphthalmia present in males with splice-site [46] or frameshift [21] variants, the present study demonstrates that this is much more likely to be a phenotypic spectrum of one unitary disease. Although some do refer to this entire disease entity as Ogden syndrome, another name could be *NAA10*-related neurodevelopmental syndrome. The latter name is longer and thus more cumbersome, whereas the name Ogden syndrome is more memorable and easier to introduce when describing the disease to non-experts. However, the longer name does follow the recently suggested dyadic nomenclature [47]. Further information about this can be found in Supplementary Discussion. The disease entity involving *NAA15* variants is referred to as *NAA15*-related neurodevelopmental syndrome.

## Supplementary information

Supplementary Info- Discussion and Figures

Supp Table 1_Human Disease Gene May 2022_filled_in_forms - HDG

Supp Table 2_NAA10 Master list DEIDENTIFIED For Journal

Supp Table 3_NAA15 Master list DEIDENTIFIED for Journal

Supp Table 4_ClinVar annotation

Supplementary_Table_5_GMDB_summary

Supplementary Table_6_ABC Vineland data

## Data Availability

Summary data is included in Supplementary information, including as excel files. Due to privacy restriction, the data are deidentified, and all additional data are available from the corresponding author on reasonable request. Facial photos are available at GestaltMatcher Database (GMDB; https://db.gestaltmatcher.org).

## Code availability

All summary data and percentages for phenotype data for these and other published (and some unpublished) *NAA10* and *NAA15* cases was curated at the Human Disease Gene website series [33], under the gene names *NAA10* and *NAA15*, at the following links using the “Graph and Chart” tab: https://humandiseasegenes.nl/naa10/ and https://humandiseasegenes.nl/naa15/.
