# Supplementary material for: Expanding the phenotypic spectrum of NAA10-related neurodevelopmental syndrome and NAA15-related neurodevelopmental syndrome
Source: Eur J Hum Genet. 2023 May 2;31(7):824–33. doi: 10.1038/s41431-023-01368-y (PMC10325952; doi:10.1038/s41431-023-01368-y)
Supplement: Supplementary file 1 — Supplementary Info- Discussion and Figures [file 41431_2023_1368_MOESM1_ESM.pdf]

Supplementary Information

Supplementary Introduction

NAA15 function has been linked to cell survival, tumor progression, and retinal development (1,2). N-terminal (Nt-) acetylation (NTA) is one of the most common protein modifications, occurring co- and post-translationally (3). Approximately 80% of cytosolic proteins are N-terminally acetylated in humans and ~50% in yeast (4), while NTA is less common in prokaryotes and archaea (3). NTA is catalyzed by a set of enzymes, the NATs, which transfer an acetyl group from acetyl coenzyme A (Ac-CoA) to the free α-amino group of a protein's N-terminus. To date, eight distinct NATs (NatA – NatH) have been identified in metazoan (NatA-F and NatH) and plant (NatG) species that are classified based on different conserved subunit compositions and substrate specificities. NTA has been implicated in steering protein folding, stability or degradation, subcellular targeting, and complex formation (5). Although NatA is not essential in *S. cerevisiae*, depletion of Naa10 or Naa15 has strong effects, including slow growth and decreased survival when exposed to various stresses.

The prospective collection of phenotyping information presented here, through a set of direct interviews with the families, has produced a better overall understanding of the range of organ systems affected and the overall natural history of these conditions. A subset of these families underwent functional assessment of the probands, with one psychologist (E.I.) administering the Vineland-3 to all patients coming to the Jervis clinic for developmental assessments.

Supplementary Methods

Clinical features

Consent forms were provided to the families for a research study collecting medical information and, for those families who provided written consent, an in-person assessment or videoconference assessment was performed with each family by G.J.L. The in-person assessments included nine visits by *NAA10* families from September 2019 to March 2020, but

these visits stopped with the onset of the COVID19 pandemic in March 2020. During these nine visits, the research participants were examined by a medical geneticist, a neurologist, a psychiatrist, and an optometrist. Phenotypic information was also obtained from clinical records with varying data available, ranging from a list of the key clinical features to detailed history and examination findings. The sequencing kits and technology varied based on the different companies involved, and the variants of interest were highlighted and verified in the collected clinical diagnostic test reports.

A separate written informed consent was obtained for publication of photographs. A summary template was formulated and filled in for each research participant, where this template was first completed by a research coordinator (E.M.) and then reviewed by a second person for accuracy. The phenotype information from all published and unpublished cases was uploaded to the Human Disease Gene website series (6) with secondary data quality control completed by a second person as well, to ensure accuracy and completeness. Human Phenotype Ontology (HPO) terms were selected in the Human Disease Gene website and the percentages represent the collation of groups of multiple terms falling under one feature archetype. For example, “Motor delay” included all probands listed as having “Gait disturbance”, “Fine Motor Delay”, “Gross Motor Delay”, “Difficulty Standing” or “Inability to stand” entered into their summary. While the intent of this method was to create a more simplified and comprehensive gestalt of the OS patient, the natural limitation to this method is the lack of nuance in distinguishing minute differences in presentation from one patient to another. Another limitation may be the overlap of symptoms falling between different feature archetypes, such as “Poor eye contact” being heavily associated with (but not exclusive to) “autistic behavior”, the latter intended to reflect more stereotyped and repetitive behavior. Future research may wish to parse out the fine distinctions between these behaviors for greater clarity. Both pertinent positive and negative features were noted, with phenotypes classified as “pertinent negatives” only if the provided clinical information explicitly stated that a phenotype was denied by the parents, was noted to be absent during physical examinations, or not reported during diagnostic procedures. Clinical features were only reported as pertinent negatives if this was explicitly

mentioned in the records, otherwise, these were classified as “unknown” or “not available”. The relative prevalence of each phenotype was calculated by dividing the number of individuals positive for the phenotype by the sample size. Percentiles for head circumference, weight and height were calculated using CDC growth charts. On the website, for *NAA10*, the protein variants were numbered with three significant digits, thus allowing sorting in order from 1 to 235 amino acids; for example, p.Arg83Cys. For *NAA15*, the cDNA and protein variants were numbered with four digits, also to allow sorting.

Variant Bioinformatics Methodology

For the annotation of ClinVar variants, the combined annotation dependent depletion (CADD) scores, MPC scores, Polyphen-2, SIFT scores, M-CAP_score and GERP++_RS were retrieved from the dbNSFP4.3a database. Consensus scoring for “pathogenic” was assigned when CADD-scores were above 23, MPC scores were>=2, GERP++_RS score were >=2, and SIFT, Polyphen and M-CAP all provided supportive evidence for pathogenicity for these variants. The variant was assigned by consensus as benign when more than 3 above approaches provided evidence of benign or tolerance. We also classified variants according to the ACMG Guidelines and the Association for Molecular Pathology (ACMG/AMP) framework (7). For gnomAD frequency, the population maximum frequency is set at 4.066e-06 in Annovar, so a period in that column (**Supplementary Table 4**) indicates that the allele is less frequent than this.

Molecular Analysis of NAA10 and NAA15 Mutations

The structures were visually inspected using PyMol, which was also used in conjunction with Adobe Illustrator CC. The discussion was also informed by numerous biochemical and biophysical studies of NAA10 and NAA15 mutant proteins (8–16).

Patient clustering analysis description

Phenotypes (manually curated HPO terms and identifiers) of patients were extracted using Perl and taken as input of clustering analysis. Resnik similarity score (17) was calculated between any

two HPO terms using the Python module ssmpy (18). Given each pair of patients, the similarity score is formalized as:

$$Sim(P_1, P_2) := avg\left(\sum_{t \in P_1} \left(max_{t' \in P_2} Sim(t, t')\right)\right)$$

where P_1 and P_2 represent patient 1 and 2, and *t* and *t'* represent HPO terms in patient 1 and 2. Similarity score matrix between patients were taken as input of software R. Patient-patient difference were calculated based on 1/Resnik similarity score. Hierarchical clustering analysis was performed using hclust function in R based on patient-patient difference. Hierarchical clustering results was visualized as dendrogram in R. Patients from NAA10 and NAA15 were highlighted using different colors. In addition to tree structure, we visualized the clustering results using multidimensional scaling (MDS) based on patient-patient difference matrix. Specifically, we assume $x_i = (x_{i1}, \dots, x_{in})$ in n-dimensional space for each patient *i* and estimate coordinate matrix $X = (x_1, \dots, x_N)$ for all patients by minimizing strain function:

$$Strain(x_1, \dots, x_N) = \left(\frac{\sum_{i,j}(b_{ij} - x_i^T x_j)^2}{\sum_{i,j} b_{ij}^2}\right)^{1/2}$$

where b_{ij} are element of the double centralized patient-patient difference matrix $B = -\frac{1}{2}(I - J_n)D_n(I - J_n)$, where I is identity matrix, J_n is all-one matrix and D_n is patient-patient distance square matrix. Then, to minimize the strain function above, singular value decomposition is applied to matrix B. Patient-coordinate matrix $X = E_m\Lambda_m^{1/2}$ where E_m is the matrix of *m* eigenvectors of *B* and Λ_m is the diagonal of *m* eigenvalue of *B*. To visualize the patient clustering results, we reduce dimension of *X* to two by selecting top two largest eigenvalue of E_m. All process is implemented in R.

Facial Gestalt Methodology

We first utilized tSNE (19) to visualize the distribution of *NAA10* and *NAA15* patients in the two-dimensional space. The information on all patients performed in this analysis is shown in **Supplementary Table 5**. The mean pairwise cosine distance between patients from the two cohorts is examined to measure the similarity. We sampled control distributions of mean pairwise

distances between two cohorts stemming a) from the same syndrome and b) from two different syndromes (shown in blue (a) and red (b) in **Supplementary Figure 8**. Cohorts were sampled from patients in the GMDB that were not included in the training of GestaltMatcher. Then, a ROC analysis was conducted to derive a threshold to decide whether two cohorts stem from the same or different syndromes. The control distributions and resulting threshold were tuned via a five-fold cross-validation resulting in a final threshold of $c = 0.9176$. To test the similarity of the two cohorts C_1 and C_2, their mean pairwise cosine distance $d(C_1, C_2)$ is calculated and compared to c. Additionally, we sample two subcohorts from C_1 and C_2 respectively and calculate their mean pairwise cosine distance for 100 times. If at least 50% of those 100 subsampled comparisons fall above the threshold c, it is considered as evidence for the two cohorts stemming from different syndromes. The approach was tested on the validation fold not used in the derivation of c comprising 66 syndromes. The method correctly detected two cohorts stemming from the same syndrome in 80.30% of the comparisons and correctly identified two cohorts stemming from different syndromes in 91.98% of the comparisons.

Rating scales

Adaptive behavior subdomains make up the most fine-grained score level. The primary norm-referenced scores for the subdomains are v-scale scores, which have a mean of 15 and standard deviation (SD) of 3. The v-scale score for each subdomain is included in the narrative interpretation. Standard scores have a mean of 100 and SD of 15. Confidence intervals reflect the effects of measurement error and provide, for each standard score, a range within which the proband's true standard score falls with a certain probability or confidence. The confidence level chosen for the report is the 90% confidence interval. A percentile rank is the percentage of individuals in the age group who scored the same or lower than the proband. For example, a percentile rank of 41 indicates that the examinee scored higher than (or the same as) 41% of the age-matched norm sample.

Supplementary Results

Subjects and Variants

An in-person assessment was performed on nine families with *NAA10* variants from October 2019 – March 2020 and an additional 26 families with *NAA10* variants were interviewed by videoconferencing from April 2020 until September 2020 due to the COVID19 pandemic. For families with *NAA15* variants, ten families were seen via videoconferencing from April 2020 until September 2020, with referral for cognitive evaluation. After this time, from October 2020 until July 2021, an additional 20 families with *NAA10* variants and 9 families with *NAA15* variants were seen via videoconferencing.

Other variants were recurrent, but at a much lower frequency, including p.Tyr43Ser, p.Ile72Tyr, p.Ala87Ser, p.Phe128Leu, and p.Met147Thr. In this cohort of individuals, novel variants include: p.His16Pro, p.Tyr31Cys, p.Ala104Asp, p.Arg116Trp, p.His120Pro, p.Leu121Val, p.Ser123Pro, p.Phe128Ser (same position as p.Phe128Leu), p.Thr152Argfs*6, and p.Glu181Alafs*67. Most of the *NAA10* variants are *de novo*, with the exception of p.Tyr43Ser in a previously reported family (20), p.Ile72Thr in two families (one of which, Individual 8, was previously reported (12,15) and Individual 54 with frameshift variant p.Thr152Argfs*6, with this same p.Thr152Argfs*6 variant being reported in two other cases (12,21). For *NAA15*, two of these individuals (4 and 19) were published previously and referred to as Individuals 14 and 21 in that paper (22). Almost all variants are *de novo*, with the exception of a splice site variant with an unknown inheritance pattern.

All families seen by September 2020 were scheduled for cognitive evaluations by one qualified psychologist (E.I.). For families with *NAA15* variants, none of the families were seen in-person prior to pandemic onset. One family with a recently deceased child with a *NAA10* variant provided extensive medical records and consent to publish but was unable to meet for videoconferencing.

World maps showing the location of the cases for *NAA10* and *NAA15* are included in the public-facing portion of the Human Disease Gene website series (6). The website series is meant to be an open-source venue for such data (6).

In regard to the entries in ClinVar, much of the information in ClinVar is de-identified, so this requires contacting the clinical testing laboratories and/or other sources of the data, and then getting referred to the clinicians who are involved in these cases. For now, it is worth at least cataloging what is currently in ClinVar, along with the new bioinformatic annotations. Many of these entries come from clinical laboratories, with very little uploaded phenotype information, and future work will involve an attempt to contact and interview these other families.

Clinical Features

The two male individuals with frameshift variants in *NAA10* (Individuals 55 and 56) are generally much higher functioning than the other individuals with missense variants. For example, Individual 56 was reported to be cognitively better than individuals his age (for example, he can count from 1 to 10 and knows almost all the letters of the alphabet and is only 29 months of age). Although it was noted that he is a couple of months behind in gross motor skills (he still cannot run or jump even though he tries. He does attend a typical nursery and the staff report that he is intelligent, and “he always wants to learn something else”. His teacher says that while she “has to explain something to individuals in his age 10 times to get them to do it, he already does it the second time”.

Facial features

Although it was previously noted that “a recognizable, regular pattern of dysmorphic facial features was not appreciated amongst the cases” (12), this is debatable now that one clinician has met and examined these individuals via videoconferencing. Eye abnormalities were found in almost all individuals, which sometimes included large, prominent eyes and large down-slanting palpebral fissures. The most common ear findings were low-set ears and large ears, with a broad range of hearing impairment and abnormalities of the outer ear. Nose features included broad nasal bridge, prominent or broad nasal tip, and anteverted or flared nares. Lastly, mouth abnormalities were present in a majority of the individuals with a high palate being the main feature, but there was a range of abnormalities in the vermillion and philtrum, as well as teeth

abnormalities with particular issues with eruption and disorganized growth. There were several cases with short chins, protruding upper lip, and microretrognathia.

For individuals with *NAA15* variants (**Supplementary Figure 5**), ,outh features were also common with abnormalities of the philtrum (long, short, broad, or smooth), teeth, upper lip vermillion, chin, and/or presence of a high palate. Abnormalities of the nose included prominent nasal tip or abnormal nasal bridge. Ear features were least common but presented mostly as outer ear abnormalities or low-set ears.

Cardiovascular features

Death can result from cardiogenic shock following arrhythmia, which was noted in some affected individuals. The arrhythmias seem to be well-controlled with pharmacotherapy in these patients and detected routinely with Holter monitor assessment. Baseline echocardiography is recommended as septal defects have been identified in several cases. Medical (pharmaceutical) management has been employed to slow the progression of congestive heart failure (CHF) when manifesting in some individuals. Electrocardiography (EKG) is also obtained at the time of diagnosis, as fatal dysrhythmias can develop with time. In several cases, the initial EKG has been normal, with evolution of arrhythmias during later periods of life. It may be necessary to evaluate on an annual basis and be vigilant for signs of and symptoms of dysrhythmia/CHF. When present, the arrhythmias have proven difficult to manage, though the full spectrum of anti-arrhythmics has not been tested. Individuals have responded to episodic cardioversion/defibrillation, but long-term treatment has not yet been determined. Pacemakers and implantable defibrillators have been used in a few cases, including in one published family (20).

Respiratory features

Individual 3 with p.Tyr31Cys missense in NAA10 died in the first year of life with local interstitial lung disease, neonatal respiratory distress, respiratory failure requiring assisted ventilation, lymphangiectasia, and pulmonary fibrosis. This included repeated apnea episodes

requiring CPR after one such episode. Radiological CT imaging of the thorax was performed at 6 weeks of age and reported “ground glass changes throughout both lungs”. The etiology remained uncertain and a lung biopsy reported mild thickening of the interstitium without significant interstitial inflammatory infiltrate, but expansion of the inter-lobular septae with lymphangiectasia and fibrosis. Focal interstitial glycogenosis was confirmed with PAS staining. The Individual 9, a male with p.Arg83Cys missense in NAA10, died around one year of age, also with respiratory arrest, neonatal respiratory distress, respiratory failure requiring assisted ventilation, tachypnea, and interstitial lung disease.

Most individuals did not have any notable respiratory issues, but a recognizable proportion of our cohort did demonstrate a pattern of recurrent respiratory infections similar to the original OS males (8), with varying severity and frequency. A larger proportion of patients with *NAA10* variants had respiratory infections as opposed to individuals with *NAA15* variants, and this pattern is replicated with the other respiratory features. A few patients also had neonatal respiratory distress, pulmonary hypertension and/or apnea (such as Individuals 12, 38, 45, 47 and 56). Given the relative lack of respiratory distress in most cases, many clinical tests, such as CT scanning and/or lung biopsy were not clinically indicated, so these data are simply not available. As such, it is not known if these individuals never had any interstitial lung disease or if they perhaps had a mild case which subsequently resolved over time. In this regard, it was written that one female with p.His16Pro in *NAA10* needed postnatally to “have an oxygen mask applied at night because of oxygen desaturations”, and “because of symptoms resembling interstitial lung disease (chronic tachy-dyspnea, recurrent pneumonia and bronchitis), a lung biopsy was performed at the age of 3 years without revealing any specific findings” (11). As such, it is possible that some of these individuals might have interstitial lung disease neonatally, but that in many cases, this might resolve over time.

Gastrointestinal recommendations

A recommendation has been made that OS individuals not tracking above the failure to thrive (FTT) range past 1 year of age should be evaluated for G-tube placement to avoid

prolonged growth failure. If G-tubes are not immediately helping to increase weight gain after insertion, recommendations include altering formula, increasing caloric input, or exchanging a G-tube for a GJ-tube by means of a minimally invasive procedure.

Growth, including height, weight and head circumference

Many of the OS individuals and a few of the individuals with *NAA15* variants have low height and weight, as detailed in a recent study (23). Poor growth cannot be entirely explained by inadequate caloric intake, inability to properly chew or swallow, growth hormone deficiency, or low appetite; past the age of 6-12 months, calorie tracking, caloric supplementation, and using feeding therapy to successfully teach individuals how to chew, swallow, and no longer choke on food did not induce adequate weight gain. Low appetite was also not a consistent cause of poor growth, as multiple proband parents claimed that their individuals were constantly hungry, and that "desire to eat has never been a problem." Effect of growth hormone (GH) administration on weight gain among GH-deficient OS individuals with growth failure should be further investigated in subsequent studies.

Ophthalmologic and visual features

CVI affects the processing of visual information, which has a profound impact on their ability to learn and must be thoroughly assessed to cater to the unique needs of each child and their impairment. Generally, most individuals have learning disabilities, and particularly seem to have difficulty with depth perception and discerning contrast, which may contribute to their difficulty using stairs or walking off of curbs. CVI was not thoroughly investigated for this paper and should be further explored with more examinations of these individuals to understand how their CVI impacts their learning and/or visuomotor abilities.

Individual 55 has p.Glu181Alafs*67 and does not have microphthalmia, indicating that even frameshift variants toward the C-terminus of NAA10 can have quite variable outcomes.

Endocrine and Metabolic features

It is up to each individual treating clinician to decide whether to pursue clinical testing to explore whether these individuals are indeed lean and/or have decreased brown adipose tissue. Brown adipose tissue amounts have not yet been assessed in affected individuals, particularly as there are logistical challenges with getting children and adults with severe intellectual disability to cooperate with imaging exams and it is very difficult to justify the use of sedation in a research setting.

Neurologic recommendations

It seems warranted to obtain brain MRI with diffusion tensor imaging, to check for white matter delays, in the first few years of life, and then a follow-up MRI around puberty. Given that some of the individuals develop seizures, it seems that baseline EEG should be obtained to check for any seizure activity and, ideally, an annual EEG, or at least one every 2-3 years, should be performed up until their early 20's. Prophylactic treatment with anti-epileptic medication should be considered if EEG shows any possible seizures. Lastly, given that some of the individuals have developed autism, it is recommended to perform standard autism screening assessments during childhood, which can include Autism Diagnostic Inventory (ADI) or Autism Diagnostic Observation Schedule (ADOS-2) screening.

Cognitive and psychiatric features

One of the males (Individual 7) with a maternally inherited missense variant (c.215T>C, p.Ile72Thr) in NAA10 had an almost normal level of functioning. However, Individual 8 (now deceased) had the exact same variant, but his ABC standard score was 59, demonstrating again that there is substantial variability even among the exact same variants. Comprehensive analyses of the many different Vineland sub-scale, age-equivalent scores (AES), and growth-scale values (GSVs) (24) falls outside the scope of the current paper and will be reported in a future paper. There are clearly other factors affecting the overall trajectory of these individuals. in addition to just the one variant they have in *NAA10* or *NAA15*, which could include, among

other things, the cardiac arrhythmias, feeding difficulties, delayed speech, delayed motor development, seizures, and cortical visual impairment.

Molecular Analysis of the Variants

The observation that frameshifts occurring at the C-terminal region of NAA10 are associated with a lower impact on cognitive function is consistent with the conserved NAA10 domain being the minimal region necessary for *in vitro* activity.

The double variant, [c.22C>T;30C>G] p.[Pro8Ser;Asp10Glu] in the exon/intron boundary, leads to a reduction in NAA10 formation by altering the splicing of the mRNA from this allele (**Supplementary Figure 1)**.

The molecular basis for the p.Asn864Ser mutant is unclear because published NatA structures are not resolved past residue 841.However, there are two published phosphorylation sites p.Ser855 and p.Ser856 in this region that have not been evaluated but may be impacted by this variant.

Supplementary Discussion

Due to a limitation in the journal for the maximum number of references allowed to be cited, we were unable to cite all relevant case reports in the main body of the paper. However, we are citing some of these additional case reports here, for *NAA10*-related neurodevelopmental syndrome (20,25–33) and *NAA15*-related neurodevelopmental syndrome(34–38).

Males with *NAA10*-related neurodevelopmental syndrome may have symptoms at different ages: those with arrhythmias or other serious cardiac conditions may be embryonic lethal; others are apparent at birth experiencing cardiac concerns, hypotonia and dysmorphic features; and others come to attention later with relatively nonspecific developmental/intellectual and growth impairments. Likewise, some females can present with similar issues as males, including rarely microphthalmia and/or prolonged QT interval, and the majority of the females

with *NAA10* variants have severe to profound intellectual disability, whereas the individuals with *NAA15* variants are much less severely impaired (see **Figure 3**).

The trajectory of these individuals seems to improve with intensive therapy, whereas some of these individuals do not make progress without adequate therapy. An area for future investigation concerns the amount of physical, speech, occupational, and other therapies that should be recommended. Based on these extensive interviews with the families, the anecdotal information seems to suggest that individuals with *NAA10*-related neurodevelopmental syndrome benefit from the following therapies on at least a weekly basis in at least the first 7 years of life, so as to ensure that they can eat, walk, and ideally verbalize: Mobility therapy; speech and language therapy; sensory integration therapy; hydrotherapy; occupational therapy with a focus on fine motor skills; and cortical visual impairment therapy (if warranted). Future studies can investigate this question much more quantitatively with a natural history study, documenting the exact modalities and frequencies of therapies.

We mentioned one limitation of our study related to the fact that only the first nine research participants were interviewed in-person and physically examined by medical doctors, including by a medical geneticist, a neurologist, a psychiatrist, and an optometrist. After this time, all interviews were converted to videoconferencing starting in March 2020, with the onset of the COVID19 pandemic, and the families met with the psychiatrist only (GJL). There is an emerging literature regarding the use of videoconferencing and telemedicine, and there are recent papers demonstrating that valuable information can still be gathered via videoconferencing (39–41).

There are multiple ophthalmologic abnormalities in these females, and individual 23 is a female with the p.Arg83Cys missense in NAA10, with microphthalmia, just like Individual 54 (a male) with p.Thr152Argfs*6 in *NAA10* also with microphthalmia, and similar in phenotype to previously published cases (12,30,42). The cognitive functioning of the females with p.Arg83Cys missense in NAA10 is also similarly impaired as the other females with different missense changes (**Figure 3**), and there is a phenotypic spectrum including multiple organ systems in all of these females with missense changes in NAA10.

Protein isolated from mammalian cell culture offers a unique advantage, where, depending on the immunoprecipitation conditions, NatA likely co-purifies with relevant binding partners, such as the NatA regulatory protein, HYPK, and the NatE catalytic subunit, NAA50. However, this does not allow for selective isolation of the NAA10 catalytic subunit or the NatA complex without either of its binding partners. By contrast, *Sf*9 overexpression of the human NatA complex can be performed with and without the co-transfection of the HYPK virus to allow for selective production of the NatA and NatA/HYPK complexes. In addition, it is also possible to prepare purified NatE complex with or without HYPK(43). However, this approach has its limitations because the Sf9-expressed NatA complex construct contains a C-terminally truncated NAA10 (1-160 of 235 total residues). This C-terminal region can be phosphorylated at 6 sites (44), and could potentially influence the activity and the stability of NAA10, although the interplay of these phosphorylation events with NAA10 and NAA15 variants has not yet been evaluated. While recombinant NAA10 overexpression in *E. coli* allows for the studying of the full-length monomeric NAA10, proteins produced in *E. coli* are not post-translationally modified.

NatA plays a complex role in the cell with a range of functional roles and varying substrate types, depending on the enzyme's oligomeric state. This is particularly apparent with the NAA10 p.Arg83Cys variant. The heterodimeric p.Arg83Cys NatA complex has enhanced activity, HYPK binding returns p.Arg83Cys NatA activity back to wild type levels (12) while the monomeric mutant NAA10 features a diminished level of activity(16). Despite this, the phenotype of the individuals with this particular mutation is not appreciably different from the other individuals reported herein. This spectrum highlights the need to build off the groundwork laid by focused biochemical studies into model systems that can account for NatA's multiple oligomeric states and cellular functions. One such multidimensional approach has been to study the human mutations in the human genes expressed in a *S. cerevisiae* model knocked out for endogenous yeast *Naa10* and/or *Naa15* (10,22,45). However, this approach has its own caveats. For example, *S. cerevisiae* is a much simpler unicellular organism that does not represent the complexity of a human cell or a human being, including the absence of expression of the NatA regulatory subunit, HYPK. Instead, these mutations are perhaps best studied using

patient-derived or cell lines with the mutations engineered into them, as has been done recently with *NAA15* (46), and/or with animal models with the human variants engineered into *NAA10* or *NAA15*.

We present in this paper some of the cardinal features that can be present in NAA10-related neurodevelopmental syndrome, including intellectual disability, seizures, cardiac anomalies, hypotonia, and a certain facial gestalt, just to name a few. We argue that there is a recognizable phenotype that emerges once enough cases have been ascertained, just like with Down Syndrome, Fragile X syndrome, Angelman syndrome, Rett Syndrome, and CDKL5 syndrome. The overall data are consistent with a phenotypic spectrum for these alleles involving multiple organ systems, including visual development. Now that one clinician has met and interviewed 61 probands and their families, it appears that this is one recognizable phenotype that has not been adequately described in the literature. Prior papers have been misleading due to not including adequate clinical information, alongside the fact that different clinicians have interviewed the families and provided retrospective information, whereas when the same clinician interviews the families, there is a recognizable condition ascertained, with a phenotypic spectrum. Although some papers have also suggested that there might be different allelic presentations for different variants involving NAA10, such as with microphthalmia present in males with splice-site(42), the overall data demonstrates that this is much more likely to be a phenotypic spectrum of one unitary disease, with decreased function of NatA being the main mechanism involved. Our paper reveals a female with Arg83 Cys variant presenting with microphthalmia and a child with a frameshift variant in Naa15 also presenting with microphthalmia, so this is a phenotypic spectrum of one disease, involving both genes that contribute to the NatA complex.

Although a prior review suggested that the name Ogden syndrome should be reserved only for the male neonatal lethal phenotype (47), this has been problematic, as there are some males that survive the neonatal period (14,20) (including Individual 7 and 54 reported herein) and many families have reported that the name Ogden syndrome is more memorable. Additionally, some clinicians have also diagnosed females as having Ogden syndrome (32). The

name, Ogden syndrome, was suggested by the first family identified, living in Ogden, Utah, when the clinician GJL visited the family and collected the DNA samples necessary to identify the genetic basis of the syndrome. The discovery process was written about previously(48). At the time that the first paper was published in 2011, it was only two families, and the consensus at that time was that it was not necessary to give a name to the condition, with only two families known. However, as other families have been identified, it seemed prudent to give it a name. The two possible options have been: Ogden syndrome and/or NAA10-related neurodevelopmental syndrome. Although we now favor the former option, it is very well possible that some in the community will just refer to it as NAA10-related syndrome, as we suggested in a prior review paper when it was not obvious to us at that time that this is a phenotypic spectrum(47). However, given that the original family wanted to call it Ogden syndrome, and given that this name is much more memorable and less cumbersome to use, we currently favor including it as an option to refer to the entire disease. In fact, the nonprofit foundation in America dedicated to this disease uses this name: https://www.ogdencares.org/

We disagree then that Ogden syndrome should be reserved for “a condition specific to the c.109T>C (p.Ser37Pro) variant", as we have come to realize that this is a condition with a phenotypic spectrum, including males who do not die from cardiac arrhythmias but rather can survive into adulthood(20). We readily acknowledge that future mechanistic work will help to reveal whether or not decreased function of NatA is the definite and primary mechanism, but the current data support this notion. However, we also note that such issues with naming tend to resolve themselves over time, as the community will eventually coalesce around what to call this. To illustrate this point, for many years, the two syndromes Fragile X and progeria, were also referred to as Martin-Bell and Hutchinson-Gilford syndromes, respectively. This issue of nomenclature is quite complex, with some people preferring to name syndromes after the first and last authors of papers, or sometimes after the clinicians involved, but in the current case, we have named the syndrome according to the wishes of the first family identified in Ogden, Utah. From our perspective, we will continue to refer to the condition as Ogden syndrome

and/or NAA10-related neurodevelopmental syndrome, and the community will eventually decide what to refer to this as, just as was done with Fragile X and progeria.

Supplementary References

1. Arnesen T, Betts MJ, Pendino F, Liberles DA, Anderson D, Caro J, et al. Characterization of hARD2, a processed hARD1 gene duplicate, encoding a human protein N-alpha-acetyltransferase. BMC Biochem. 2006;7:13.

2. Gendron RL, Laver NV, Good WV, Grossniklaus HE, Miskiewicz E, Whelan MA, et al. Loss of tubedown expression as a contributing factor in the development of age-related retinopathy. Invest Ophthalmol Vis Sci. 2010 Oct;51(10):5267–77.

3. Dörfel MJ, Lyon GJ. The biological functions of Naa10 - from amino-terminal acetylation to human disease. Gene. 2015;567(2):103–31.

4. Arnesen T, Van Damme P, Polevoda B, Helsens K, Evjenth R, Colaert N, et al. Proteomics analyses reveal the evolutionary conservation and divergence of N-terminal acetyltransferases from yeast and humans. Proc Natl Acad Sci U S A. 2009 May 19;106(20):8157–62.

5. Aksnes H, Ree R, Arnesen T. Co-translational, Post-translational, and Non-catalytic Roles of N-Terminal Acetyltransferases. Mol Cell. 2019 Mar 21;73(6):1097–114.

6. Dingemans AJM, Stremmelaar DE, Vissers LELM, Jansen S, Nabais Sá MJ, van Remortele A, et al. Human disease genes website series: An international, open and dynamic library for up-to-date clinical information. Am J Med Genet A. 2021 Apr;185(4):1039–46.

7. Richards S, Aziz N, Bale S, Bick D, Das S, Gastier-Foster J, et al. Standards and guidelines for the interpretation of sequence variants: a joint consensus recommendation of the American College of Medical Genetics and Genomics and the Association for Molecular Pathology. Genet Med. 2015 May;17(5):405–24.

8. Rope AF, Wang K, Evjenth R, Xing J, Johnston JJ, Swensen JJ, et al. Using VAAST to identify an X-linked disorder resulting in lethality in male infants due to N-terminal acetyltransferase deficiency. Am J Hum Genet. 2011;89(1):28–43.

9. Myklebust LM, Van Damme P, Stove SI, Dörfel MJ, Abboud A, Kalvik TV, et al. Biochemical and cellular analysis of Ogden syndrome reveals downstream Nt-acetylation defects. Hum Mol Genet. 2015;24(7):1956–76.

10. Van Damme P, Støve SI, Glomnes N, Gevaert K, Arnesen T. A Saccharomyces cerevisiae model reveals in vivo functional impairment of the Ogden syndrome N-terminal acetyltransferase NAA10 Ser37Pro mutant. Mol Cell Proteomics. 2014 Aug;13(8):2031–41.

11. Bader I, McTiernan N, Darbakk C, Boltshauser E, Ree R, Ebner S, et al. Severe syndromic ID and skewed X-inactivation in a girl with NAA10 dysfunction and a novel heterozygous de novo NAA10 p.(His16Pro) variant - a case report. BMC Med Genet. 2020 Jul 22;21(1):153.

12. Cheng H, Gottlieb L, Marchi E, Kleyner R, Bhardwaj P, Rope AF, et al. Phenotypic and biochemical analysis of an international cohort of individuals with variants in NAA10 and NAA15. Hum Mol Genet [Internet]. 2019 May 25; Available from: http://dx.doi.org/10.1093/hmg/ddz111

13. McTiernan N, Gill H, Prada CE, Pachajoa H, Lores J, CAUSES study, et al. NAA10 p.(N101K) disrupts N-terminal acetyltransferase complex NatA and is associated with developmental delay and hemihypertrophy. Eur J Hum Genet. 2021 Feb;29(2):280–8.

14. McTiernan N, Tranebjærg L, Bjørheim AS, Hogue JS, Wilson WG, Schmidt B, et al. Biochemical analysis of novel NAA10 variants suggests distinct pathogenic mechanisms involving impaired protein N-terminal acetylation. Hum Genet [Internet]. 2022 Jan 17; Available from: http://dx.doi.org/10.1007/s00439-021-02427-4

15. Støve SI, Blenski M, Stray-Pedersen A, Wierenga KJ, Jhangiani SN, Akdemir ZC, et al. A novel NAA10 variant with impaired acetyltransferase activity causes developmental delay, intellectual disability, and hypertrophic cardiomyopathy. Eur J Hum Genet. 2018 Sep;26(9):1294–305.

16. Saunier C, Stove SI, Popp B, Gerard B, Blenski M, AhMew N, et al. Expanding the Phenotype Associated with NAA10-Related N-Terminal Acetylation Deficiency. Hum Mutat. 2016 Aug;37(8):755–64.

17. Resnik P. Using information content to evaluate semantic similarity in a taxonomy [Internet]. arXiv [cmp-lg]. 1995. Available from: http://arxiv.org/abs/cmp-lg/9511007

18. Couto FM, Lamurias A. Semantic Similarity Definition. In: Encyclopedia of Bioinformatics and Computational Biology. Elsevier; 2019. p. 870–6.

19. Gmail L@., Hinton G. Visualizing Data using t-SNE [Internet]. 2008 [cited 2023 Feb 6]. Available from: https://www.jmlr.org/papers/volume9/vandermaaten08a/vandermaaten08a.pdf?fbcl

20. Casey JP, Støve SI, McGorrian C, Galvin J, Blenski M, Dunne A, et al. NAA10 mutation causing a novel intellectual disability syndrome with Long QT due to N-terminal acetyltransferase impairment. Sci Rep. 2015 Nov 2;5:16022.

21. Shishido A, Morisada N, Tominaga K, Uemura H, Haruna A, Hanafusa H, et al. A Japanese boy with NAA10-related syndrome and hypertrophic cardiomyopathy. Hum Genome Var. 2020 Aug 17;7(1):23.

22. Cheng H, Dharmadhikari AV, Varland S, Ma N, Domingo D, Kleyner R, et al. Truncating Variants in NAA15 Are Associated with Variable Levels of Intellectual Disability, Autism Spectrum Disorder, and Congenital Anomalies. Am J Hum Genet. 2018;102(5):985–94.

23. Sandomirsky K, Marchi E, Gavin M, Amble K, Lyon GJ. Phenotypic variability and Gastrointestinal Manifestations/Interventions for growth in Ogden syndrome (also known as NAA10-related Syndrome) [Internet]. medRxiv. 2022. Available from: http://medrxiv.org/lookup/doi/10.1101/2022.03.16.22272517

24. Farmer CA, Kaat AJ, Thurm A, Anselm I, Akshoomoff N, Bennett A, et al. Person Ability Scores as an Alternative to Norm-Referenced Scores as Outcome Measures in Studies of Neurodevelopmental Disorders. Am J Intellect Dev Disabil. 2020 Nov 1;125(6):475–80.

25. Maini I, Caraffi SG, Peluso F, Valeri L, Nicoli D, Laurie S, et al. Clinical Manifestations in a Girl with NAA10-Related Syndrome and Genotype–Phenotype Correlation in Females. Genes . 2021 Jun 10;12(6):900.

26. Gogoll L, Steindl K, Joset P, Zweier M, Baumer A, Gerth-Kahlert C, et al. Confirmation of Ogden syndrome as an X-linked recessive fatal disorder due to a recurrent NAA10 variant and review of the literature. Am J Med Genet A. 2021 Aug;185(8):2546–60.

27. Afrin A, Prokop JW, Underwood A, Uhl KL, VanSickle EA, Baruwal R, et al. NAA10 variant in 38-week-gestation male patient: a case study. Cold Spring Harb Mol Case Stud [Internet]. 2020 Dec;6(6). Available from: http://dx.doi.org/10.1101/mcs.a005868

28. Ree R, Geithus AS, Tørring PM, Sørensen KP, Damkjær M, DDD study, et al. A novel NAA10 p.(R83H) variant with impaired acetyltransferase activity identified in two boys with ID and microcephaly. BMC Med Genet. 2019 Jun 7;20(1):101.

29. Gupta AS, Saif HA, Lent JM, Couser NL. Ocular Manifestations of the NAA10-Related Syndrome. Case Rep Genet. 2019 Apr 8;2019:8492965.

30. Johnston JJ, Williamson KA, Chou CM, Sapp JC, Ansari M, Chapman HM, et al. NAA10 polyadenylation signal variants cause syndromic microphthalmia. J Med Genet. 2019 Mar 6;jmedgenet-2018.

31. Valentine V, Sogawa Y, Rajan D, Ortiz D. A case of de novo NAA10 mutation presenting with eyelid myoclonias (AKA Jeavons syndrome). Seizure. 2018 Aug;60:120–2.

32. Sidhu M, Brady L, Tarnopolsky M, Ronen GM. Clinical Manifestations Associated With the N-Terminal-Acetyltransferase NAA10 Gene Mutation in a Girl: Ogden Syndrome. Pediatr Neurol. 2017 Nov;76:82–5.

33. Popp B, Stove SI, Endele S, Myklebust LM, Hoyer J, Sticht H, et al. De novo missense mutations in the NAA10 gene cause severe non-syndromic developmental delay in males and females. Eur J Hum Genet. 2015 May;23(5):602–9.

34. Straka I, Švantnerová J, Minár M, Stanková S, Zech M. Neurodevelopmental gene-related dystonia-parkinsonism with onset in adults: A case with NAA15 variant. Mov Disord. 2022 Sep;37(9):1955–7.

35. Yubero D, Martorell L, Nunes T, Lyon GJ, Ortigoza-Escobar JD. Neurodevelopmental gene-related dystonia: A pediatric case with NAA15 variant. Mov Disord. Wiley; 2022 Nov;37(11):2320–1.

36. Tian Y, Xie H, Yang S, Shangguan S, Wang J, Jin C, et al. Possible Catch-Up Developmental Trajectories for Children with Mild Developmental Delay Caused by NAA15 Pathogenic Variants. Genes [Internet]. 2022 Mar 18;13(3). Available from: http://dx.doi.org/10.3390/genes13030536

37. Ritter A, Berger JH, Deardorff M, Izumi K, Lin KY, Medne L, et al. Variants in NAA15 cause pediatric hypertrophic cardiomyopathy. Am J Med Genet A. 2021 Jan;185(1):228–33.

38. Stessman HA, Xiong B, Coe BP, Wang T, Hoekzema K, Fenckova M, et al. Targeted sequencing identifies 91 neurodevelopmental-disorder risk genes with autism and developmental-disability biases. Nat Genet [Internet]. 2017; Available from: https://www.ncbi.nlm.nih.gov/pubmed/28191889

39. Shah AC, Badawy SM. Telemedicine in pediatrics: Systematic review of randomized controlled trials. JMIR Pediatr Parent. 2021 Feb 24;4(1):e22696.

40. Levy T, Lerman B, Halpern D, Frank Y, Layton C, Zweifach J, et al. CHAMP1 disorder is associated with a complex neurobehavioral phenotype including autism, ADHD, repetitive behaviors and sensory symptoms. Hum Mol Genet. 2022 Aug 17;31(15):2582–94.

41. St John M, Amor DJ, Morgan AT. Speech and language development and genotype-phenotype correlation in 49 individuals with KAT6A syndrome. Am J Med Genet A. 2022 Dec;188(12):3389–400.

42. Esmailpour T, Riazifar H, Liu L, Donkervoort S, Huang VH, Madaan S, et al. A splice donor mutation in NAA10 results in the dysregulation of the retinoic acid signalling pathway and causes Lenz microphthalmia syndrome. J Med Genet. 2014 Jan 15;51(3):185–96.

43. Deng S, McTiernan N, Wei X, Arnesen T, Marmorstein R. Molecular basis for N-terminal acetylation by human NatE and its modulation by HYPK. Nat Commun. 2020 Feb 10;11(1):818.

44. Olsen JV, Vermeulen M, Santamaria A, Kumar C, Miller ML, Jensen LJ, et al. Quantitative phosphoproteomics reveals widespread full phosphorylation site occupancy during mitosis. Sci Signal. 2010 Jan 12;3(104):ra3.

45. Dörfel MJ, Fang H, Crain J, Klingener M, Weiser J, Lyon GJ. Proteomic and genomic characterization of a yeast model for Ogden syndrome. Yeast. 2017 Jan;34(1):19–37.

46. Ward T, Tai W, Morton S, Impens F, Van Damme P, Van Haver D, et al. Mechanisms of congenital heart disease caused by NAA15 haploinsufficiency. Circ Res. 2021 Apr 16;128(8):1156–69.

47. Wu Y, Lyon GJ. NAA10-related syndrome. Exp Mol Med. 2018;50(7):85.

48. Lyon GJ. Personal account of the discovery of a new disease using next-generation sequencing. Interview by Natalie Harrison. Pharmacogenomics. 2011 Nov;12(11):1519–23.

Supplementary Tables as excel files

Supplementary Table 1_Human Disease Gene May 2022_filled_in_forms - HDG - NAA10 and NAA15

Supplementary Table 2_NAA10 Master list DEIDENTIFIED For Journal

Supplementary Table 3_NAA15 Master list DEIDENTIFIED for Journal

Supplementary Table 4_ClinVar annotations

Supplementary Table 5: Summary of patients used in the GestaltMatcher analysis. The images can be accessed via the GMDB ID column.

Supplementary Table_6_ABC Vineland data DEIDENTIFIED for journal

Supplementary Figures

A)

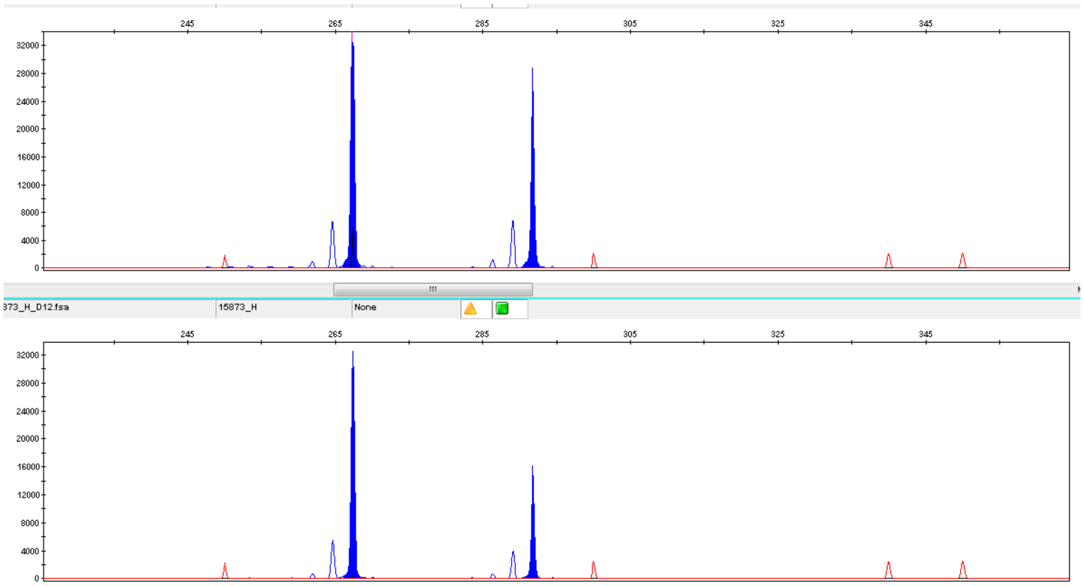

B)

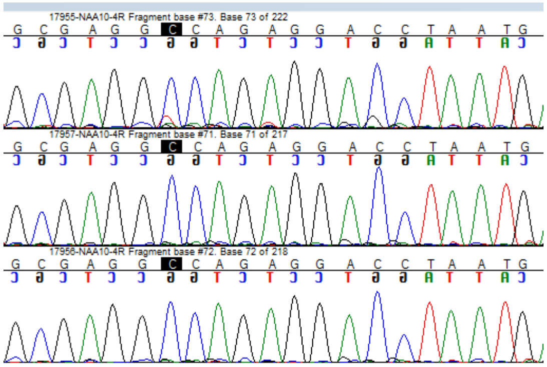

C)

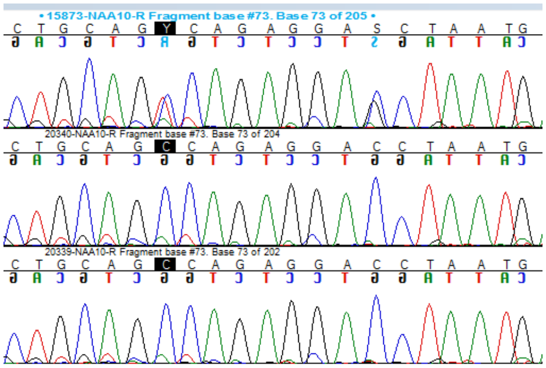

Supplementary Figure 1. Complementary analyses in individual 1. (A) X chromosome inactivation pattern assessed by the androgen receptor (AR) methylation assay [Allen et al., 1992] with minor modifications. Note that both X-chromosomes show a pattern of random inactivation in a ratio 60:40 (B) cDNA Sanger sequencing of exons 1 to 4 of *NAA10* gene from the patient (upper row) and her parents shows an almost exclusive expression of the normal allele. (C) DNA Sanger sequencing of exon 2 (*NAA10* gene) confirms *de novo* c.22C>T and c.30C>G variants.

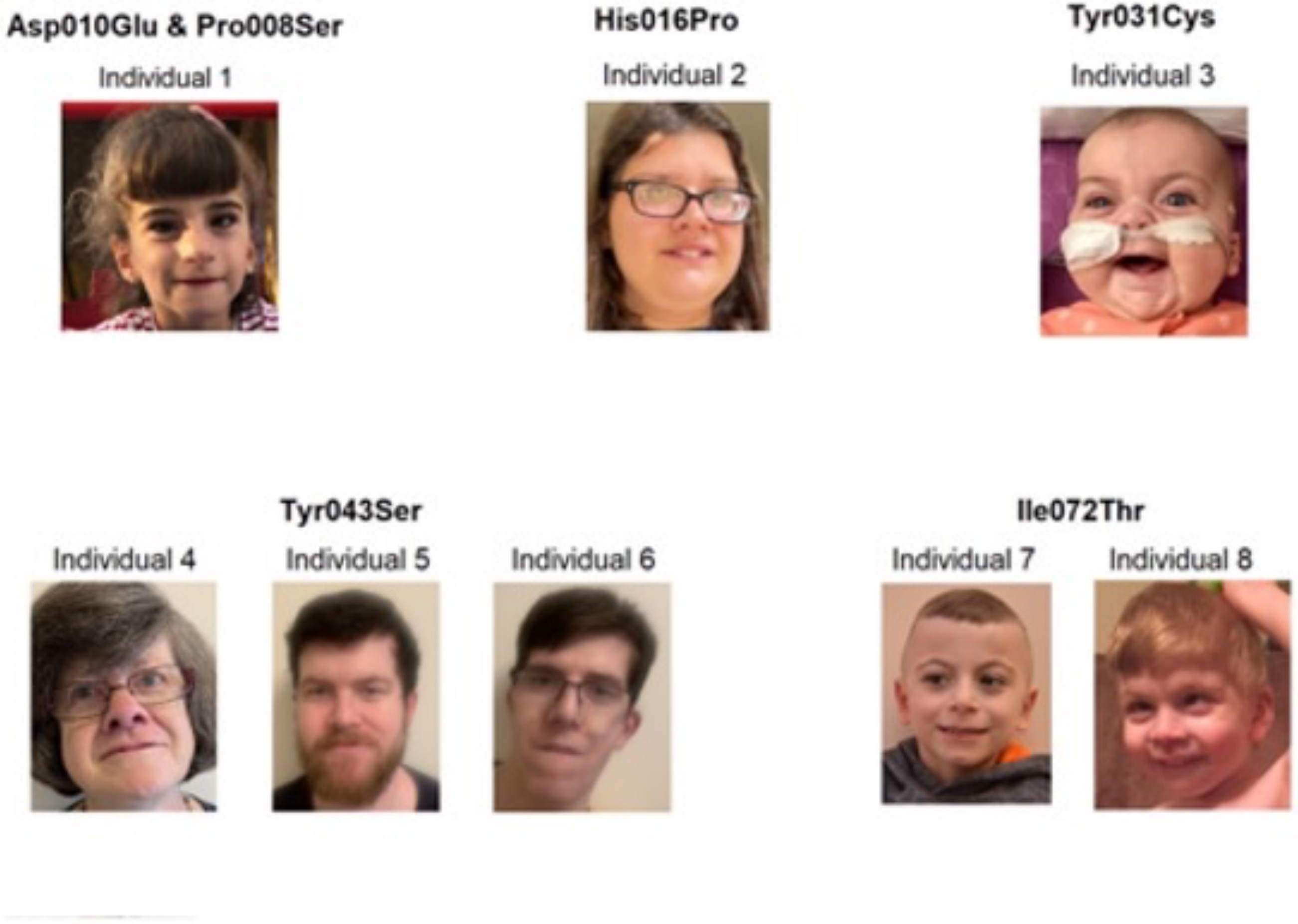

Supplementary Figure 2.

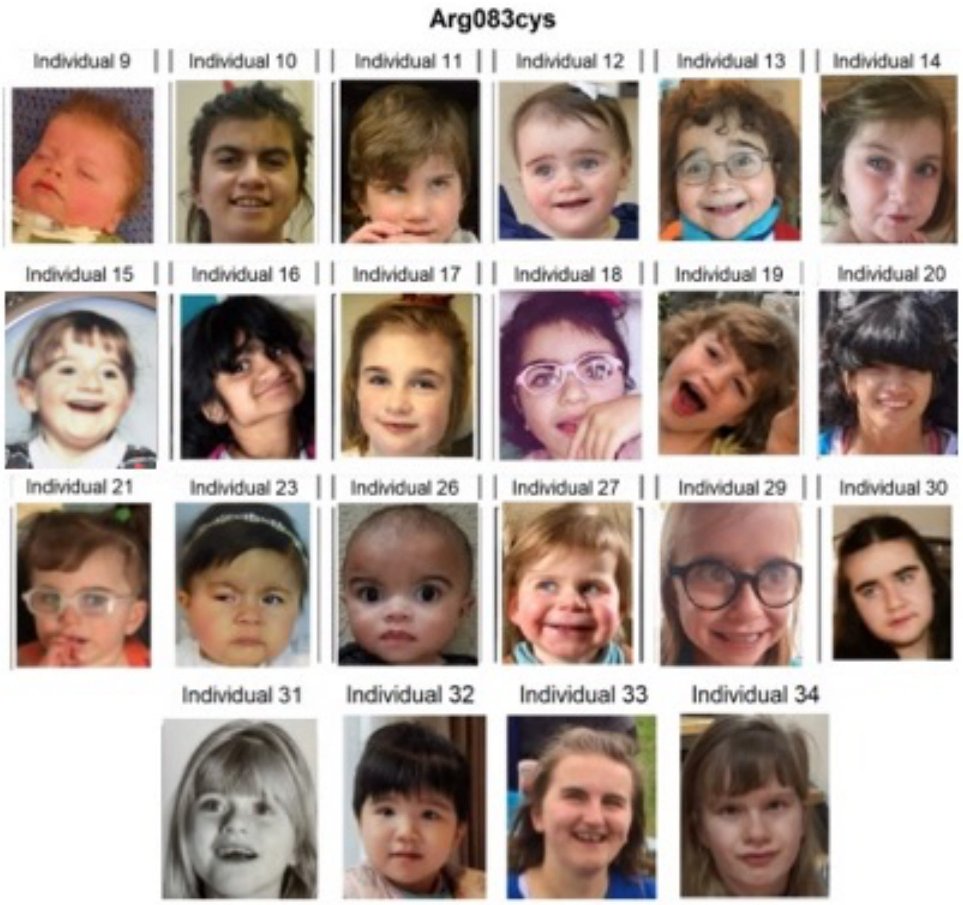

Supplementary Figure 3.

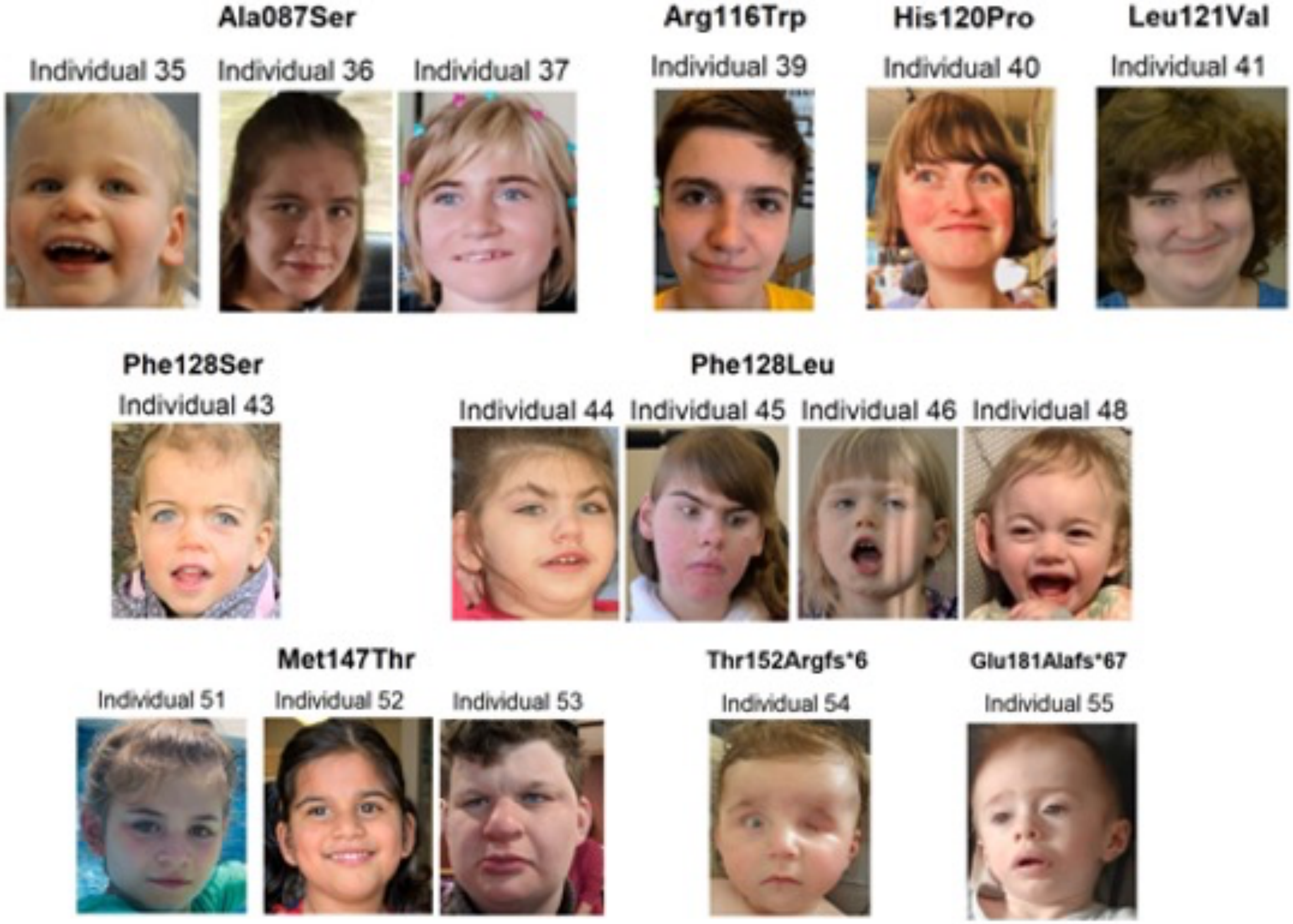

Supplementary Figure 4.

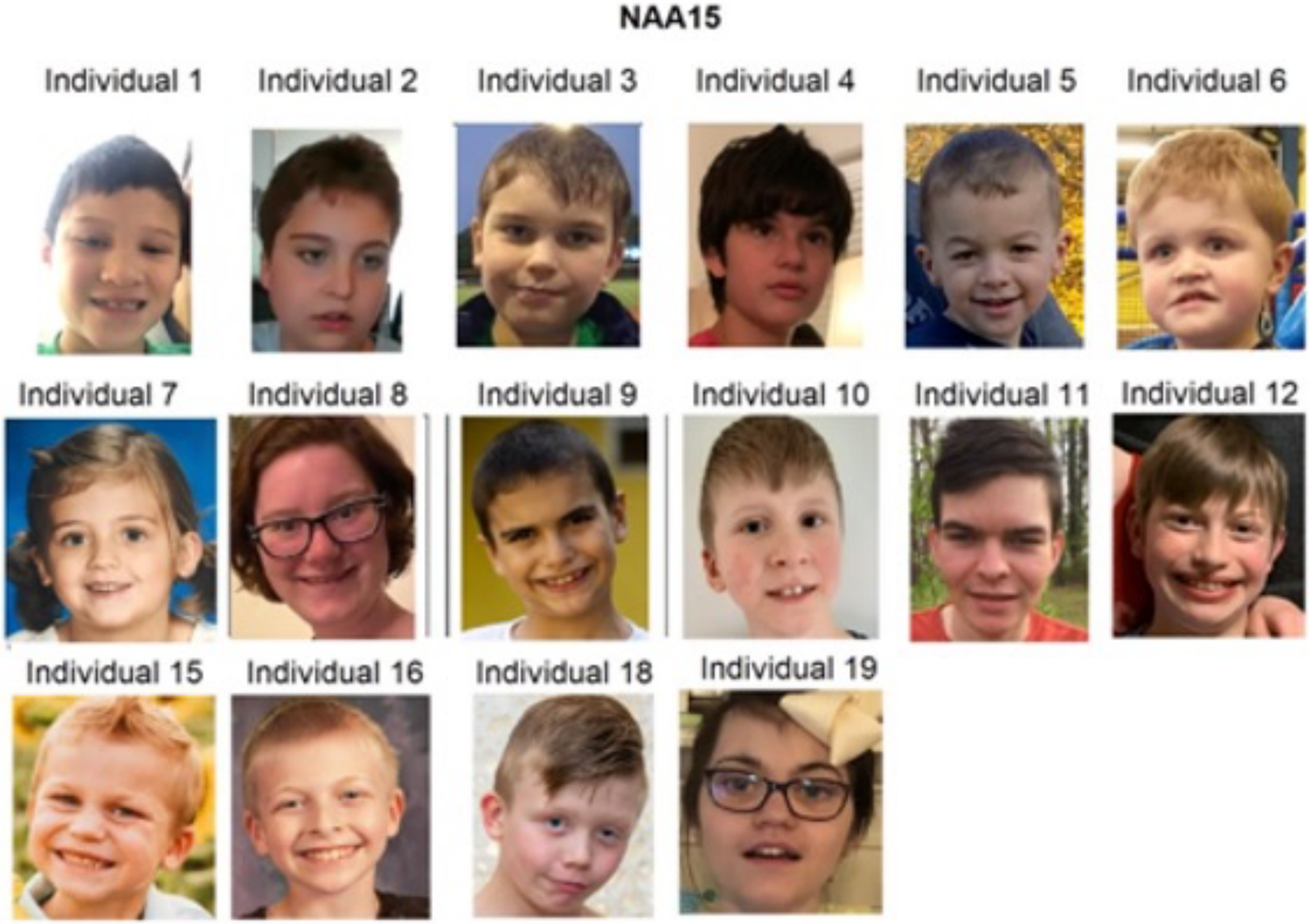

Supplementary Figure 5.

A)

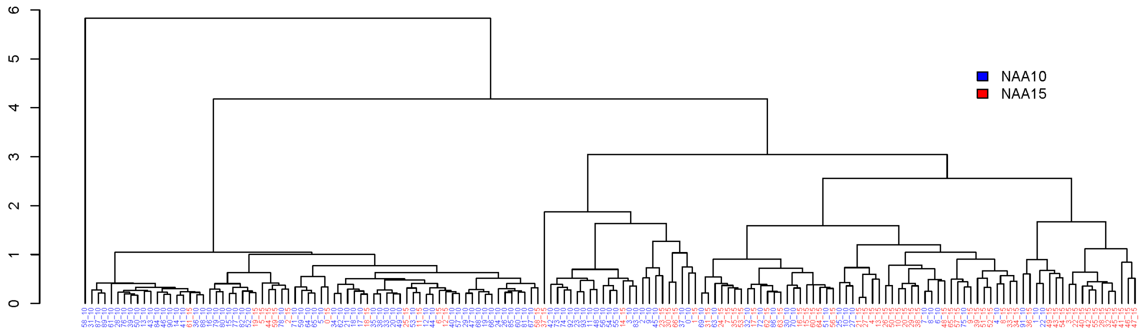

B)

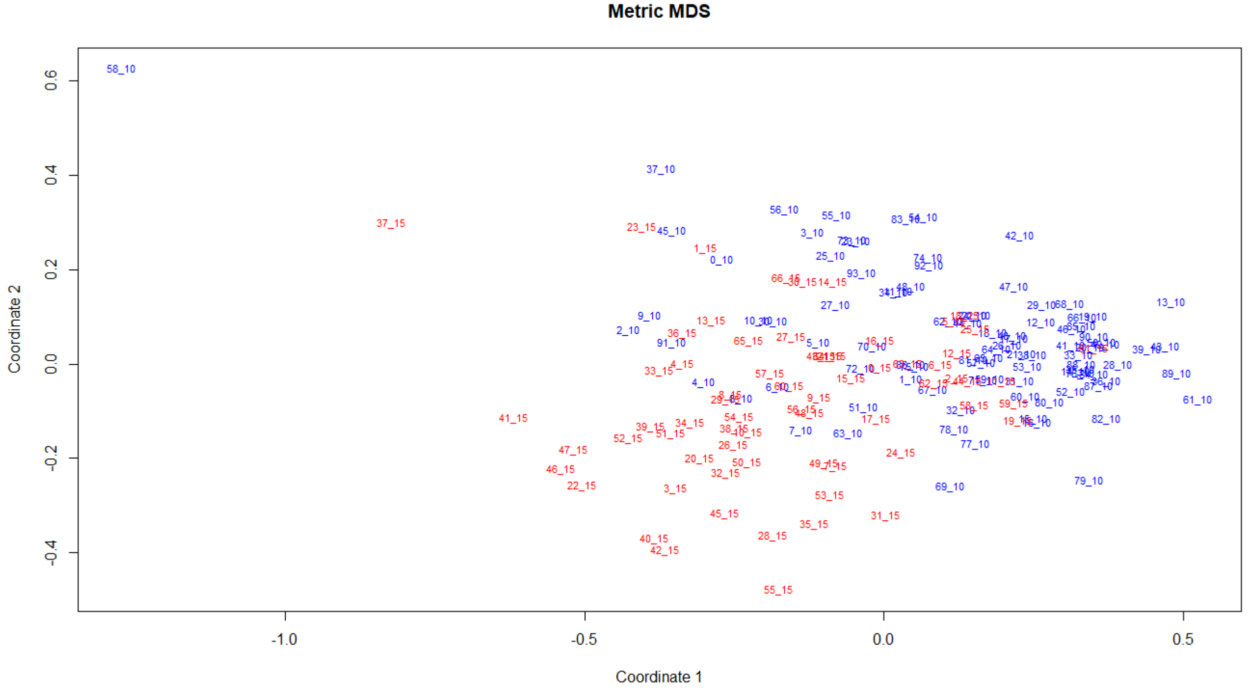

Supplementary Figure 6. Visualization of NAA10/15 patient clustering results. (A) Dendrogram showing NAA10/15 patient classification results (blue for NAA10 and red for NAA15). The distance metric between patients is based on HPO term similarity score. (B) Scatter plot of patients based on multidimensional scaling (MDS). Each blue dot represents one NAA10 patient and each red dot represents one NAA15 patient.

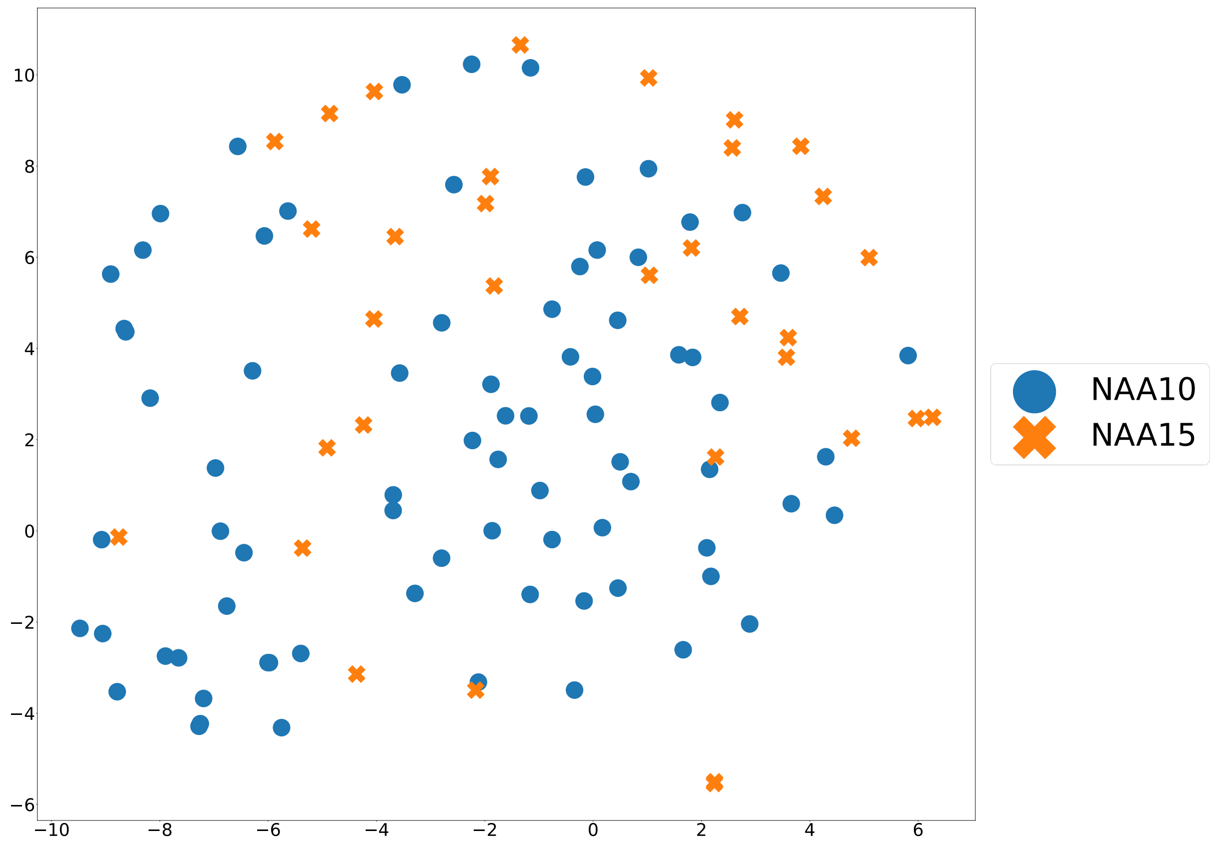

Supplementary Figure 7: tSNE visualization of NAA10 and NAA15 patients.

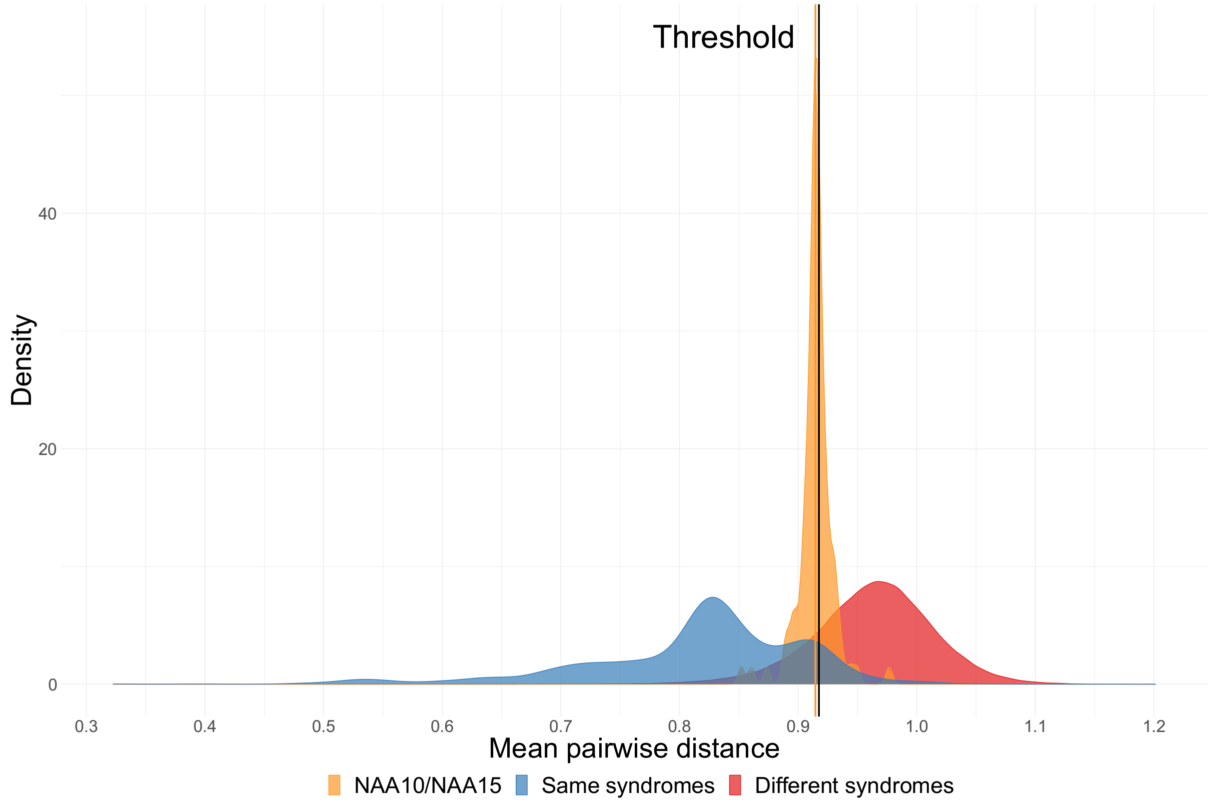

Supplementary Figure 8: Mean pairwise distance distribution of cohorts sampled from (blue) same syndrome, (red) different syndrome, and (orange) NAA10 and NAA15. Threshold (c) is 0.9176. The mean pairwise distance between NAA10 and NAA15 patients is 0.9146, and 67% of the sampling is below the threshold (the region falling on the left of the threshold).
